# *Actinobacillus pleuropneumoniae* genes expression in biofilms cultured under static conditions and in a drip-flow apparatus

**DOI:** 10.1186/1471-2164-14-364

**Published:** 2013-05-31

**Authors:** Yannick DN Tremblay, Vincent Deslandes, Mario Jacques

**Affiliations:** 1 Groupe de recherche sur les maladies infectieuses du porc, Faculté de médecine vétérinaire, Université de Montréal, 3200 Sicotte, St-Hyacinthe, Québec, J2S 7C6, Canada

**Keywords:** *Actinobacillus pleuropneumoniae*, Biofilm, Transcriptome, Drip-flow apparatus

## Abstract

**Background:**

*Actinobacillus pleuropneumoniae* is the Gram-negative bacterium responsible for porcine pleuropneumonia. This respiratory infection is highly contagious and characterized by high morbidity and mortality. The objectives of our study were to study the transcriptome of *A. pleuropneumoniae* biofilms at different stages and to develop a protocol to grow an *A. pleuropneumoniae* biofilm in a drip-flow apparatus. This biofilm reactor is a system with an air-liquid interface modeling lung-like environment. Bacteria attached to a surface (biofilm) and free floating bacteria (plankton) were harvested for RNA isolation. Labelled cDNA was hybridized to a microarray to compare the expression profiles of planktonic cells and biofilm cells.

**Results:**

It was observed that 47 genes were differentially expressed (22 up, 25 down) in a 4 h-static growing/maturing biofilm and 117 genes were differentially expressed (49 up, 68 down) in a 6h-static dispersing biofilm. The transcriptomes of a 4 h biofilm and a 6 h biofilm were also compared and 456 genes (235 up, 221 down) were identified as differently expressed. Among the genes identified in the 4 h vs 6h biofilm experiment, several regulators of stress response were down-regulated and energy metabolism associated genes were up-regulated. Biofilm bacteria cultured using the drip-flow apparatus differentially expressed 161 genes (68 up, 93 down) compared to the effluent bacteria. Cross-referencing of differentially transcribed genes in the different assays revealed that drip-flow biofilms shared few differentially expressed genes with static biofilms (4 h or 6 h) but shared several differentially expressed genes with natural or experimental infections in pigs.

**Conclusion:**

The formation of a static biofilm by *A. pleuropneumoniae* strain S4074 is a rapid process and transcriptional analysis indicated that dispersal observed at 6 h is driven by nutritional stresses. Furthermore, *A. pleuropneumoniae* can form a biofilm under low-shear force in a drip-flow apparatus and analyses indicated that the formation of a biofilm under low-shear force requires a different sub-set of genes than a biofilm grown under static conditions. The drip-flow apparatus may represent the better *in vitro* model to investigate biofilm formation of *A. pleuropneumoniae*.

## Background

*Actinobacillus pleuropneumoniae* is the Gram-negative bacterium responsible for porcine pleuropneumonia. This severe and highly contagious infectious respiratory disease causes major economic losses in the swine industry [1,2]. Transmission is by means of aerosol or by direct contact with infected animals and the infection may result in rapid death or in severe pathology [1]. Animals exposed to *A. pleuropneumoniae* may develop chronic infections or become asymptomatic carriers that may transmit the disease to healthy herds [1]. The virulence factors involved in colonization and induction of lung lesions, which include type IV fimbriae, lipopolysaccharides (LPS) and the pore forming RTX toxins ApxI to IV, have been well characterized (for a recent review see [2]). The role of biofilms in *A. pleuropneumoniae* pathogenicity is gaining recognition.

Biofilm formation is involved in the virulence of numerous bacterial pathogens including those of veterinary importance [3]. Biofilms are defined as structured communities of bacterial cells enclosed in a self-produced matrix attached to biotic or abiotic surfaces [4]. The ability to form a biofilm is considered an universal trait of microorganisms. Furthermore, biofilms offer protection against hostile environments, the immune response and bactericidal concentration of antibiotics or disinfectants. *A. pleuropneumoniae* has the ability to form biofilms under certain static growth conditions [5,6] and several field isolates of *A. pleuropneumoniae* can form biofilms. For *A. pleuropneumoniae*, biofilm formation on polystyrene microtiter plates depends on the production of a polymer of β-1,6-N-acetyl-D-glucosamine (PGA) [7,8]. The histone-like protein H-NS represses the expression of the *pgaABCD* operon and the alternative sigma factor σ^E^ up-regulates the expression of the operon [9]. A H-NS, an autotransporter serine protease AasP and a LuxS mutants formed more biofilms whereas a response regulator ArcA mutant was unable to form biofilms [10-13]. Furthermore, the ClpP protease has been recently associated with biofilm formation [14]. Additionally, our laboratory recently used transposon mutagenesis to identify 16 unique genetic determinants associated with biofilm formation in *A. pleuropneumoniae*[15]. The screen identified genes such as *potD2*, *ptsI*, *tig* and *rpmF*, which have all been previously associated with biofilm formation in other bacterial species. Furthermore, novel genetic determinants were identified. In a recent study, we also demonstrated that the biofilms of *A. pleuropneumoniae* field isolates were more resistant than their planktonic counterpart to ampicillin, florfenicol, tiamulin and tilmicosin [16]. Despite recent advancements, knowledge regarding the processes involved in biofilm formation of *A. pleuropneumoniae* is limited. The transcriptome of heterogeneous population of *Eshecrichia coli* biofilms have been analyzed and such analyses have provided solid insights into the biofilm lifestyle of *E. coli*[17]. Microarray technology has been used by our laboratory to characterize *A. pleuropneumoniae* grown under *in vitro* conditions mimicking steps of the infectious process [6,18-20] and during a natural infection [21].

Thus, the objectives of this study were to characterize the transcriptome of planktonic cells and biofilm cells cultured at difference stages of biofilm formation cycle and to develop a protocol to grow an *A. pleuropneumoniae* biofilm under low-shear force in a drip-flow apparatus, a system with an air-liquid interface that can model environments such as the lungs [22].

## Results and discussion

### Biofilm formation of *A. pleuropneumoniae* is a rapid process

**Figure 1:**
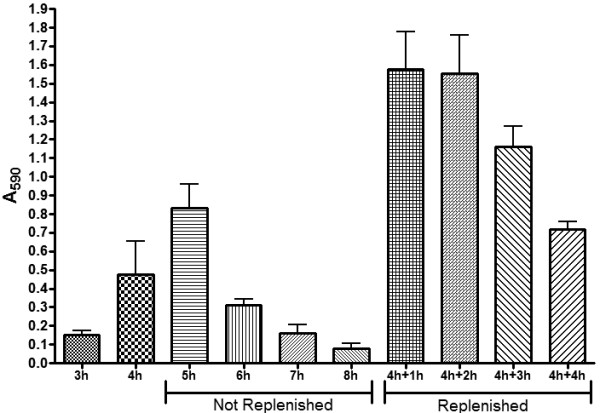
**Effect of time and medium replenishment on biofilm formation by *A. pleuropneumoniae *S4074 in microtiter plates.** Spent growth medium was removed after 4 h of incubation and fresh BHI-NAD was added to the biofilms. Biomass were then measured after 1 h (4 h+1 h), 2h (4 h+2 h), 3 h (4 h+3 h) and 4 h (4 h+4 h). The values are the average of three biological replicates and the error bar represents the standard deviation.

It was previously observed that biofilm formation in *A. pleuropneumoniae* is a rapid process [6]. To ensure that biofilm cells were harvested at the most appropriate time for the transcriptional analysis, a biofilm time course was performed to study the biofilm cycle of *A. pleuropneumoniae* strain S4074. Biomass was detectable after 3 h and was at its maximum after 5 h (Figure 1). Surprisingly, the biofilm had started to disperse after 6 h and a minimum amount of biomass was detected after 8 h (Figure 1). It was hypothesized that the dispersal was either caused by a nutritional stress or the accumulation of a dispersal signal. To test this hypothesis, the spent growth medium was removed after 4 h and fresh BHI-NAD was added to the biofilms. It was observed that changing the growth medium increased biomass and delayed the dispersal by 1 h (Figure 1). This suggested that a continuous flow of fresh nutrient or the removal of a dispersion signal is required to maintain the biofilm of *A. pleuropneumoniae* strain S4074.

**Figure 2:**
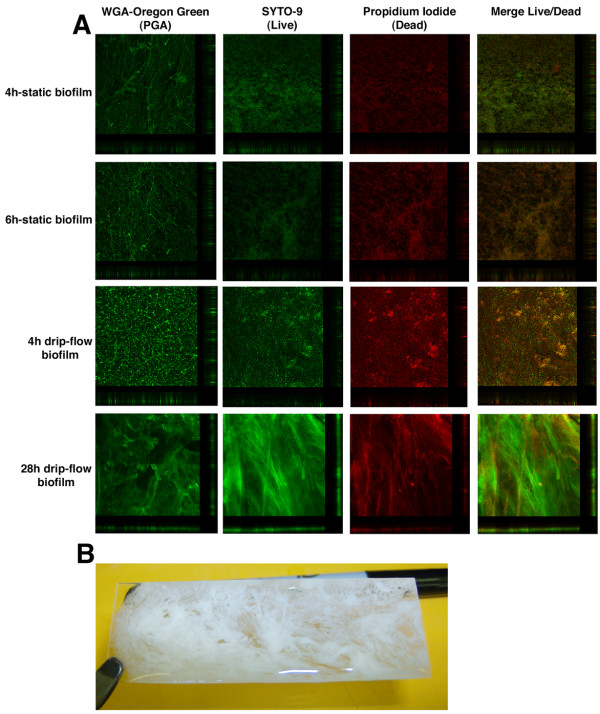
**Images of typical static and drip-flow biofilms of *A. pleuropneumoniae *S4074.** Confocal microscopy of *A. pleuropneumoniae* S4074 4 h-static biofilm and 6 h-static biofilm cultured in a microtiter plate and 4 h biofilm and 28 h biofilm cultured in a drip flow apparatus (**A**), and a typical biofilm visible after 28 h in the drip-flow apparatus (**B**). For CLSM, biofilms were stained with the SYTO-9 (live) and propidium iodide (dead) or WGA-conjugated to Oregon green and visualized at 40X for the 4 h and 6 h biofilms, and at 4X for the 28 h drip-flow biofilm.

To further characterize the biofilm process, 4 h and 6 h biofilms were analyzed by confocal laser scanning microscopy (CLSM). For analysis, biofilms were stained with WGA-Oregon green to characterize the matrix, and SYTO-9 and propidium iodide to characterize the live cell population and dead cell population, respectively. For all 3 stains, a decrease in biomass and change in the morphology of the biofilm were observed at 6 h (Figure 2A). To complement these observations, images on the Z-axis plane were acquired to generate 3D-images of 4 h and 6 h biofilms. Using the 3D-images, data concerning the volume and height of the biofilms were generated and biomass was calculated from the volume and area. At both time points, the biomass of the live cells within a biofilm was larger than the biomass occupied the dead cells. Overall, the volume and biomass of a 4 h biofilm is larger than a 6 h biofilm (Table 1). Although these difference between a 4 h and 6h biofilm were consistent in the same experiment, the variation from day to day was too great for the difference to be statistically significant. Similar variation is observed when biofilm biomass is measured with crystal violet (Figure 1). Thus, the biomass changes observed with CLSM does correlate with the changes observed with crystal violet.

**Table 1:** **Total volume, biomass and height of different *A. pleuropneumoniae *S4074biofilms as determined by CLSM and image analysis**

**Biofilm**	**Total volume (μm**^**3**^**)**	**Biomass (μm**^**3**^**/μm**^**2**^**)**	**Height**	
			**Average (μm)**	**Maximum (μm)**
Live (SYTO-9)				
Microtiter 4 h	866286 ±240194	8.58 ±2.38	3.99 ±0.44	41.06 ±1.63
Microtiter 6 h	627207 ±80602	6.21 ±0.8	4.58 ±1.26	41.43 ±2.49
Drip flow 4 h^a^	199781 ±13424	1.98 ±0.13	3.71 ±0.03	40.07 ±2.04
Drip flow 28 h^b^	900785208 ±51873026	89.21 ± 5.13	49 ±8.33	304.33 ±18.8
Dead (Propidium iodide)				
Microtiter 4 h	620814 ±148835	6.15 ±1.47	3.43 ±0.21	38.70 ±1.43
Microtiter 6 h	540838 ±146407	5.36 ±1.45	3.53 ±0.31	40.02 ±3.7
Drip flow 4 h	241597 ±99038	2.39 ±0.98	3.42 ±0.13	40.61 ±2.84
Drip flow 28 h	742364385 ±89082908	73.52 ±8.82	39.49 ±6.91	302.47 ±20.79
Live-Dead Colocalization				
Microtiter 4 h	435442 ±164297	4.31 ±1.63	4.47 ±0.44	36.10 ±1.56
Microtiter 6 h	359642 ±123216	3.56 ±1.22	4.61 ±0.94	37.66 ±3.76
Drip flow 4 h	96354 ±28557	0.95 ±0.28	2.95 ±0.11	36.98 ±4.32
Drip flow 28 h	480477866 ±93441665	47.58 ±9.25	43.29 ± 7.39	252.39 ± 23.7
WGA-Oregon Green				
Microtiter 4 h	627544 ±182810	6.21 ±1.81	3.74 ±0.89	33.45 ±6.89
Microtiter 6 h	541858 ±145811	5.37 ±1.44	4.22 ±1.8	25.78 ±6.86
Drip flow 4 h	560824 ±54936	5.55 ±0.54	5.59 ±0.87	45.88 ±0.55
Drip flow 28 h	552328813 ±52239741	54.70 ± 5.17	42.01 ±3.02	273.41 ±14.03

^a^This represents the biofilm formed during the 4 h pre-incubation prior to the initiation of the flow of fresh medium.

^b^Images were obtained with a 4X objective rather than a 40X objective.

For transcriptional analysis, it was decided that microtiter-plate biofilms would be harvested after 4 h and 6h of incubation which correspond to growth/maturation and dispersal phases of the biofilm. Furthermore, it was decided that the development of a continuous-flow system was required to study biofilm over a longer period of time.

### *A. pleuropneumoniae* forms a large and stable biofilm in a drip-flow apparatus

To study biofilm formation over a longer time period, the properties of several systems were examined. The drip-flow apparatus was selected because it is thought to create an environment with air-liquid interface that closely resembles the lung environment [22], the natural environment of *A. pleuropneumoniae*. Furthermore, *A. pleuropneumoniae* is able to form a biofilm at the air-liquid interface in a glass tube [15]. In the drip-flow protocol, a static incubation is required to ensure that the bacteria are attached to the coupon before the flow is initiated. A 4 h-static incubation prior to starting the flow was selected given that biofilm formation was at its maximum between 4 h and 5 h in our microtiter assay. For the selection of a coupon to support biofilm development, the ability of *A. pleuropneumoniae* to form a biofilm after 4 h on different materials was investigated. After a 4 h of incubation in a Lab-Tek chamber, *A. pleuropneumoniae* was able to form a biofilm on a glass slide but was not able to form a biofilm on permanox or borosilicate cover slip (data not shown). The number of CFU and the overall biofilm morphology of *A. pleuropneumoniae* cultured in a drip-flow apparatus were also determined after a 4h static incubation. The number of CFU per chamber was 10^9^ (data not shown) and the biofilm appeared as thin layers with microcolonies (Figure 2A). The volume and biomass of live and dead cells in a 4 h biofilm cultured in a drip-flow apparatus was smaller than those of a microtiter plate biofilm (Table 1). Despite the difference in the amount of live and dead cells, the biomass of the matrix as assessed with WGA was equal to the microtiter conditions (Table 1). Based on the observations mentioned above, it was concluded that a 4 h static incubation was sufficient for a biofilm to form on the glass slide before the flow was started.

To determine the “drip flow” condition, solutions of diluted BHI-NAD were assessed for their ability to support the growth of *A. pleuropneumoniae* and for their inability to disperse a pre-formed biofilm after 30 min. The 50% BHI-NAD condition did support growth and did not disperse the biofilm after 30 min. Therefore, it was concluded that a 50% BHI-NAD solution would be used in the fresh medium reservoir and a flow of 200 mL/hour per chamber would be used. After a 4 h static incubation, the flow was initiated and left for 24 h. The number of CFU and the overall biofilm morphology was then determined. The number of CFU increased from 10^9^ to 10^10^ per chamber after 24 h of flow and the biofilm was visible with the naked eye (Figure 2B). The size of the biofilm makes microscopy analysis difficult. Nevertheless, the biofilm could be stained with WGA indicating the presence of PGA in the matrix and the biomass of live cells was larger than the biomass of dead cells (Figure 2B). Furthermore, treatment with dispersin B dispersed the biofilm suggesting that PGA is also the major component of a 28 h-drip flow biofilm (data not shown). This is the first report that described the formation of a biofilm by *A. pleuropneumoniae* under low shear force and in a continuous flow system.

### Genes expressed under static biofilm conditions

**Figure 3:**
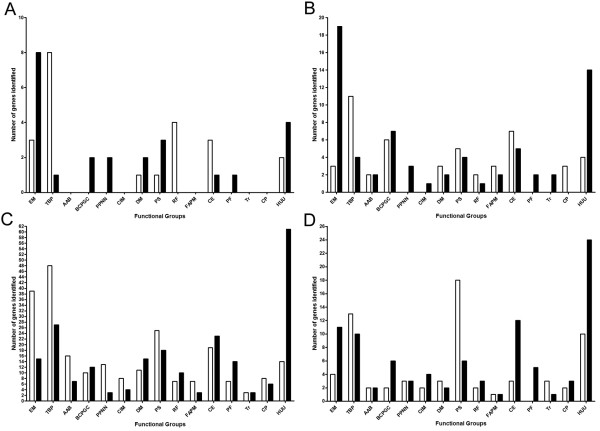
**Functional classification of the differentially expressed genes of *A. pleuropneumoniae *S4074 in static and drip-flow biofilms.** Bar charts represents the 4 h static biofilm (**A**), the 6 h static biofilm (**B**), the 4 h vs 6 h static biofilm experiment (**C**) the 28 h drip-flow biofilm (**D**). The white and black columns represent up-regulated and down-regulated genes, respectively. AAB, amino acid biosynthesis. BCPGC, biosynthesis of cofactors, prosthetic groups and carriers. CE, cell enveloppe. CP, cellular process. CIM, central intermediary metabolism. DM, DNA metabolism. EM, energy metabolism. FAPM, fatty acid and phospholipid metabolism. HUU, hypothetical/unclassified/unknown. MEE, mobile extrachromosomal element functions. PF, protein fate. PS, protein synthesis. PPNN, purines, pyrimidins, nucleosides and nucleotides. RF, regulatory functions. ST, signal transduction. Tr, transcription. TBP, transport and binding proteins.

At 4 h, 47 genes were differentially expressed (22 up, 25 down) between the biofilm cells and the planktonic cells (Table 2 and Additional file 1: Table S2). Based on their functional classification, the majority of energy metabolism genes identified as differentially expressed were down-regulated in the biofilm and represented the largest group of down-regulated genes (Figure 3A). Among those, subunits for two key enzymes for anaerobic metabolism were identified: glycerol-3-phosphate dehydrogenase (APL_0379 and APL_0381) and formate dehydrogenase (APL_0894 and APL_0895). The largest group of up-regulated genes were transport-related genes followed by genes associated with regulatory functions (Figure 3A). Among the transport-related genes, two homologues for the transport of key metabolite in the energy metabolism were identified: a C4-dicarboxylate transporter (DcuB) and a glycerol-3-phosphate transporter (GlpT). One gene worth mentioning is the gene encoding the sensor histidine kinase, CpxA. It has been demonstrated that the CpxRA regulates genes involved in biofilm-formation in *E. coli* strain MC4100 [23]. Additionally, several genes with unknown role or uncharacterized function were down-regulated in the biofilm.

**Table 2:** ***A. pleuropneumoniae *S4074 genes of interest that were differentially expressed in biofilms**

**Locus tag**	**Gene**	**Conditions**							
		**4 h-static biofilm**		**6 h-static biofilm**		**4 h vs 6 h static biofilms**		**Drip-flow biofilm**	
		**Up**	**Down**	**Up**	**Down**	**Up**	**Down**	**Up**	**Down**
APL_0048	*arcA*						√		
APL_0049							√		
APL_0189	*dus*		√				√	√	
APL_0234									√
APL_0236							√		√
APL_0330							√		√
APL_0331	*hlp*								√
APL_0364	*ssa1 /aasP*						√		
APL_0379	*glpA*		√						
APL_0381	*glpC*		√						
APL_0382	*ribD*			√		√		√	
APL_0383	*ribE*				√	√			
APL_0384	*ribA*				√				
APL_0391	*macA*						√		√
APL_0394	*rpoE*						√		√
APL_0395	*rseA*						√		
APL_0443							√		
APL_0449						√		√	
APL_0626	*macB*						√		√
APL_0627	*cpxA*	√							
APL_0629	*cpxR*								√
APL_0840	*tolC*						√		√
APL_0891	*fdhD*						√		
APL_0892	*fdxG*				√	√			
APL_0893	*fdxG*				√	√			
APL_0894	*fdxH*		√		√	√			
APL_0895	*fdnI*		√		√	√			
APL_0896	*fdhE*		√		√				
APL_0936								√	
APL_0959							√		
APL_1045							√		√
APL_1110								√	
APL_1159							√		√
APL_1387				√		√		√	
APL_1494	*ftpA*								√
APL_1550	*wecD*					√			
APL_1552	*wecB*					√			
APL_1553						√			
APL_1554	*wecA*			√		√			
APL_1674	*dmsA*				√	√			
APL_1675	*dmsB*				√	√			
APL_1676	*dmsC*				√	√			
APL_1875							√		√
APL_1921	*pgaA*						√		
APL_1922	*pgaB*						√		
APL_1923	*pgaC*						√		
APL_1924	*pgaD*						√		
APL_1957							√		√
APL_1965	*crp*						√		
APL_2012								√	
APL_2029								√	

At 6 h, 117 genes were differentially expressed (49 up, 68 down) between the biofilm cells and the planktonic cells (Table 2 and Additional file 1: Table S3). Again, the largest group of down-regulated genes and up-regulated genes were energy-metabolism genes and transport-related genes, respectively (Figure 3B). Again, subunits of the formate dehydrogenase were down-regulated in the biofilm (APL_0892, APL_0893, APL_0894, APL_0895 and APL_0896). Additionally, another key component of anaerobic metabolism, which is the anaerobic dimethyl sulfoxide reductase chain (APL_1674, APL_1675, APL_1676), was down-regulated in biofilm cells. A homologue for the transport of a key metabolite in the energy metabolism was also identified: a formate transporter (FocA). Again, most genes that were classified in the hypothetical/unclassified/unknown class were down-regulated in the biofilm.

Surprisingly, few genes were differentially expressed in a 4 h-static biofilm (47 genes) and a 6 h-static biofilm (117 genes) when compared to their planktonic counterpart; this represents approximately 2.3% and 5.8% of the genome. Most of the differences between the static-biofilm and planktonic cells are related to the down-regulation of energy metabolism indicating that biofilm cells are less metabolically active as highlighted above. Furthermore, up-regulation of the transport function in the biofilm cells suggests that this population relies on import of metabolites for their metabolic needs. Based on the transcriptomic analysis, biofilm formation in *A. pleuropneumoniae* is probably not solely regulated at the transcriptional level and different post-transcriptional regulation mechanisms are probably involved. Post-transcriptional regulation of biofilms and its development has been reported for matrix polysaccharides of *Pseudomonas aeruginosa*[24] and *Staphylococcus epidermidis*[25]. Furthermore, activity of cell-surface enzymes might be regulated by environmental factors and inside-out signaling without affecting transcription. Recently, Newell et al. [26] demonstrated a transcription-independent mechanism that regulates biofilm attachment in *Pseudomonas fluorescens* by environmental phosphate and inside-out signaling. Overall, the planktonic cells rely on anaerobic metabolism for their energy requirement. Furthermore, the down-regulation of energy metabolism-associated genes and the up-regulation of transport-related genes in biofilms suggest that biofilm cells have a reduced energy metabolism and might rely upon the importation of nutrient for their metabolic needs.

### Stress-related genes are down-regulated in a growing biofilms

Given that the biofilm was in its detaching phase at 6 h, the transcriptomes of a 4 h static biofilm and a 6 h static biofilm were also compared to identify probable causes of the dispersal. A total of 456 genes (235 up, 221 down) were identified as differentially expressed (Table 2 and Additional file 1: Table S4). Based on their functional classification, the largest up-regulated groups were genes associated with transport and energy metabolism (Figure 3C). The 4 h biofilm was metabolic more active than the 6h biofilm because several anaerobic and glucose metabolism were identified. These include the formate dehydrogenase subunits (*fdxG*, *fdnI*, *fdxH*), anaerobic dimethyl sulfoxide reductase (*dmsB*) 1-phosphofructokinase (*fruK*), and glycerol kinase (*glpK*). The 4 h biofilm cells also up-regulate genes transporter for key metabolite in the energy metabolism such as two anaerobic C4-dicarboxylate transporter (*dcuB*) and two glycerol-3-phosphate transporter (*glpT*). Furthermore, the 4 h-biofilm cells appear to be an iron-rich environment given that several genes identified previously were down-regulated [18]. These include the transferrin-binding protein gene (*tbpB*), the ferric uptake regulator gene (*fur*), ferritin-like protein gene (*ftnB*), haemoglobin-binding protein gene (*hgbA*), and the TonB energy system genes (*exbD2*, *exbB2* and *tonB2*). The largest group of down-regulated genes was ORFs with unknown role or uncharacterized functions. Interestingly, three stress regulators, *arcA*, *crp* and σ^E^, were down-regulated in a 4 h static biofilm (Figure 3C; Additional file 1: Table S4). Furthermore, 58 members of the *arcA* regulon identified by Buettner, et al. [27] were differentially expressed in 4 h biofilm. The *pgaABCD* were also down-regulated in a 4 h static biofilm but this differential expression is probably controlled by σ^E^. This alternative sigma factor has been shown to control *pgaABCD* expression [9] and thus our data confirm their observation. Another gene of particular interest, APL_0443, was also down-regulated in the 4h biofilm. This gene is predicted to be an autotransporter adhesin. The down-regulation of APL_0364 is also interesting because it encodes the autotransporter serine protease AasP. An *aasP* mutant was unable to form biofilm [13]. Interestingly, the *wecABD* (APL_1552, APL_1554, APL_1550) and *wzz* (APL_1553) genes were up-regulated at 4h and these genes encode proteins involved in biosynthesis of complex carbohydrates such as bacterial common antigen and O-antigens. WecA has been shown to play a role in the biofilm formation of nontypeable *Haemophilus influenzae*[28].

Overall, the functional profile of genes identified as differentially expressed suggest that the biofilm at 4 h is healthier and more active metabolically. Furthermore, it indicates that the dispersion observed in a 6 h biofilm is driven by stress regulators such as ArcA, Crp and σ^E^. The stress initiating the dispersion has yet to be identified but based on the stress regulators, it likely related to a nutritional deficiency in the growth medium. Alternatively, a dispersal signal maybe amplified in a closed system.

### Genes expressed in drip-flow apparatus

Bacteria from biofilms cultured using the drip-flow apparatus differentially expressed 161 genes (68 up, 93 down) when compared to the effluent bacteria (Table 2 and Additional file 1: Table S5). The largest group of up-regulated genes were those associated with protein synthesis (Figure 3D). As observed with static biofilms, one of the major functional category up-regulated in the biofilm was transport-related genes and the largest group of genes down-regulated were those associated with energy metabolism (Figure 3D). Additionally, the majority of cell envelope associated genes and ORFs classified as hypothetical/unclassified/unknown were down-regulated in the biofilm. Of particular interest, several membrane protein genes were up-regulated in the biofilm (5 genes: APL_0449, APL_0936, APL_1110, APL_2012, APL_2029) but several lipoprotein genes were down-regulated (7 genes: APL_0234, APL_0236, APL_0330, APL_0331, APL_1045, APL_1159, APL_1875, APL_1957). Furthermore, the CpxR response regulator gene (APL_0629) was down-regulated in the biofilm. The CpxAR two-component system has been associated with regulating the cell envelope composition and biofilm formation [23,29]. The alternative sigma factor, σ^E^, was also down-regulated in a drip-flow biofilm; the expression of its cognate anti-factor, *rseA*, was unchanged. Expression of *pgaABCD* is controlled by σ^E^ as mentioned before [9].

Another study has also used microarray technology to dissect the biofilm life style of another bacterium, *P. aeruginosa,* in a drip-flow assay [30]. Unlike our study, genes were ranked against markers for particular physiological activities. This allowed the researchers to infer the physiological state of the biofilms and they concluded that the biofilm bacteria were glucose nourished, iron repleted, oxygen limited, and growing slowly or in a stationary-like phase [30]. Given that there is a lack of transcription study on the physiological activities of *A. pleuropneumoniae*, it is difficult to make such inferences with our functional classification and cross-referencing analyses. However, the biofilm population appeared to be growing slowly given that energy metabolism-associated genes are down-regulated. Metabolism-associated genes and transport-related genes and protein-synthesis genes have also been previously reported as the major down-regulated and up-regulated functional classes in studies relying on microarray hybridizations to characterize gene expression in biofilm population of *Porphyromonas gingivalis*[31] and *Streptococcus pyogenes*[32]. This indicates that *A. pleuropneumoniae* biofilms behave, in a general sense, similarly to those of other bacterial species. Overall, this suggests that biofilm cells have a reduced energy metabolism and might rely upon the importation of nutrient for their metabolic needs; however, the bacteria within the biofilm appear to be highly active in synthesizing proteins.

### Validation of microarray results by qRT-PCR

To confirm results obtained using the microarrays, five up-regulated genes and five down-regulated genes per experiment were selected for qRT-PCR analysis (Additional file 1: Table S1). The selected genes represented a wide array of log2 ratio and in every cases, qRT-PCR results validated the microarray results.

### Cross-referencing between biofilm conditions

**Figure 4:**
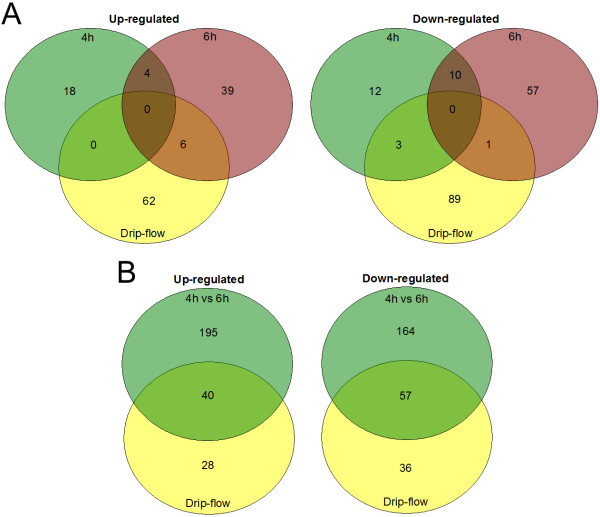
**Venne’s diagram of the *A. pleuropneumoniae *S4074 genes differentially expressed in biofilms.** (**A**) Genes differentially expressed in a 4 h and 6 h static biofilms were cross-referenced with those of 28 h drip-flow biofilm. (**B**) Genes differencially expressed in a 28 h drip-flow biofilm were cross-referenced with those identified in the 4 h static biofilm vs 6 h static biofilm assay. Genes were only considered shared between two conditions only if the genes were differentially expressed in the same direction.

Genes differentially expressed in biofilms were cross-referenced to identify genes that were shared among different biofilm conditions (Figure 4). Surprisingly, no genes were shared between all three biofilm conditions (4 h static, 6 h static, 28 h drip-flow). Both 4 h static and 6 h static biofilm (4 up, 10 down) had more in common than the drip flow biofilm and the 4 h static biofilm (0 up, 3 down) or 6 h static biofilm (6 up, 1 down). Most genes that were shared among biofilm conditions encoded proteins with unknown role or uncharacterized functions. One set of genes worth noting are the formate dehydrogenase subunit genes, APL_0894 and APL_0895, that were down-regulated in 4 h and 6 h static biofilms. The lack of commonality between biofilms might be due to the fact that each biofilms are in different phase of the biofilm development; the 4 h static biofilm is in the growing phase, the 6 h static biofilm is in the detaching phase and the 28 h drip-flow biofilm is a mature biofilm. The lack of commonality could also be explained by the difference in growth media (BHI vs 50% BHI), atmospheric conditions (5% CO_2_ vs O_2_) and the substrates (polystyrene vs glass) between the static biofilms and the drip-flow biofilms.

When the genes identified in the drip-flow experiment were compared to those of the 4 h vs 6 h static biofilm experiment, several differentially express genes were shared between both conditions (40 up, 57 down; Figure 4B). Interestingly, two of the stress-related regulators (*arcA* and *crp*) identified in the 4 h vs 6 h experiment were not differentially expressed in the drip-flow experiment. Furthermore, some genes that were up-regulated in both conditions are ribosomal proteins and a subset of the genes associated with energy metabolism. The majority of the genes that were down-regulated in both conditions encoded proteins with unknown role or uncharacterized functions. However, several of the lipoproteins identified previously were down-regulated in both experiments. Furthermore, 3 other genes (APL_0391, APL_0626, APL_0840) are worth noting because they encode components of an ABC-type transporter; APL_0391, APL_0626 and APL_0840 encode a MacA-like protein, MacB-like protein, a TolC-like protein, respectively. An ABC transporter has been implicated in the secretion of a dispersin in enteroaggregative *E. coli* (EAEC) [33]. This dispersin is a secreted 10.2-kDa protein encoded by *aap* (anti-aggregation protein) that promotes the dispersal of EAEC from surface such as glass and epithelial cells. Furthermore, MacA and MacB have been implicated in the secretion of putisolvin in *Pseudomonas putida*[34]. Putisolvin is a lipopeptide with surface-tension-reducing ability that can inhibit and disperse biofilms of *Pseudomonas* species. Overall, this suggests that effluent bacteria from the drip-flow are differently expressing genes related to a naturally detaching biofilm. Therefore, a sub-population of the biofilm formed in a drip-flow apparatus appears to undergo a process similar to seeding dispersal. This phenomenon is described as the active detachment process in biofilms that differs from erosion detachment which is passive and shear-mediated [35].

Our laboratory recently identified several genetic determinants associated with biofilm formation [15] and these determinants were cross-referenced with genes identified in this transcriptomic study. A total of 4 genetic determinants were identified: APL_0049, APL_0189, APL_0384 and APL_1387. Of the 4 genes, APL_0049 and APL_0384 are the most interesting. APL_0049 was down-regulated in the 4 h vs 6h experiment and was also down-regulated in the presence of SJPL cells [19] and during the acute phase of a natural infection [21]. Furthermore, an APL_0049 transposon mutant forms more biofilm than the parental strain [14]. Based on previous observation, we hypothesized that the expression of APL_0049 is detrimental to the attachment of *A. pleuropneumoniae*[15]. The down-regulation of APL_0049 in a growing biofilm fits this hypothesis. APL_0384 and its neighbour gene APL_0383, were down-regulated in a 6 h biofilm and a APL_0384 mutant formed less biofilm than the parental strain [15]. In contrast, APL_0382 was up-regulated in the 6 h static biofilm, in the 4 h vs 6 h experiment and the drip-flow biofilm. All 3 genes encode enzymes involved in riboflavin synthesis. The over-expression of genes associated with riboflavin synthesis during biofilm formation of *Shewanella oneidensis* has been noted before [36]. Based on these observations, it is reasonable to suggest that the riboflavin synthesis pathway might be important for biofilm formation in *A. pleuropneumoniae*.

As indicated above, only 4 out of the 16 genetic determinants identified in a transposon mutant screen were differentially expressed in our biofilm conditions. Additionally, H-NS or *luxS* were not identified as differentially expressed in our transcriptonic study, and *arcA*, *aasP*, *pgaABCD* and σ^E^ were down-regulated in a growing static biofilm (4 h) when compared to a dispersing static biofilm (6 h). The absence of several genetic determinants previously identified is puzzling but it is very likely that these determinants play a transient role in biofilm formation or that mutation in those genes have down-stream effect on biofilm formation. The down-regulation of *arcA*, *pgaABCD,* the anti-sigma E, *rseA*, and σ^E^ was also surprising given that *arcA* and *pgaC* mutants failed to form biofilms and over-expression σ^E^ increases biofilm formation [7,9,10]. The down-regulation of *aasP* (APL_0364), however, fits with previous observation such as its down-regulation in the presence of SJPL cells [19] and a *aasP* mutant adhered more to polystyrene [13]. Therefore, it is likely *aasP* interfere with adhesion and that the roles *arcA* and σ^E^ in biofilm formation are more transient. Furthermore, the expression of *pgaABCD* might be regulated at post-transcriptional level. For example, *pgaABCD* transcript in *E. coli* is targeted by RNA-binding protein CsrA and, consequently, increasing the activity of the promoter but reducing PGA production [37].

To identify genes that might be involved in host-pathogen interactions and biofilm formation, transcriptomes from this study were cross-referenced with previous transcriptional study performed by our and other laboratories [19,21,38]. The drip-flow biofilm was the condition that shared the most genes with a natural infection analysis (20 genes; Table 3; [21]) and an experimental infection analysis (13 genes; Table 3; [38]). The 4h-static biofilm and the 6h-static biofilm shared 4 and 14 genes with the natural infection analysis and shared 4 and 8 genes with the experimental infection analysis. Given that the drip-flow transcriptome shared the most genes with the natural and experimental infections transcriptome, this biofilm reactor might be the best model to study biofilm formation of *A. pleuropneumonia in vitro*. However, there was a poor relationship between the up-regulated and down-regulated genes in the biofilm conditions and the natural infections condition. For example, no genes were up-regulated in the drip-flow biofilm and the natural infection and 5 genes were down-regulated in both conditions. There was a better relationship between the genes identified in the transcriptome of *A. pleuropneumoniae* attached to SJPL cells and biofilm formation. The 4 h-static biofilm, the 6 h-static biofilm and the drip-flow biofilm shared 9 (1 up, 6 down), 14 (2 up, 10 down) and 13 (4 up, 5 down) differentially-expressed genes with the bacteria attached to SPJL cells. These included the formate dehydrogenase subunit genes, APL_0893, APL_0894 and APL_0895, and these genes were among the genes down-regulated in the static biofilms and cells attached to SJPL cells. The growth condition for the static biofilm and the attachment to SJPL cells share some similarities such as an atmosphere supplemented in 5% CO2 and the lack of agitation. Furthermore, both the adhesion assay (3 h) and the biofilm assay (4 h and 6 h) are done over a short period of time. These similarities could explain some of the commonality between the transcriptomes. Interestingly, it can, however, indicate that attachment to an abiotic (polystyrene) or a biotic surface (SJPL cells) initiate similar changes.

**Table 3:** **List of genes that were differentially expressed in biofilms and *in vivo***

***In vivo *experiments [Ref]**	**4 h-static biofilm**		**6 h-static biofilm**		**Drip-flow biofilm**	
	**Up**	**Down**	**Up**	**Down**	**Up**	**Down**
Experimental Infections [38]						
APL_0322	√					
APL_0375						√
APL_0486		√		√		
APL_0575			√			
APL_0668		√				√
APL_0740				√	√	
APL_0771		√		√		
APL_0971				√		
APL_1169					√	
APL_1285						√
APL_1379						√
APL_1385			√			
APL_1597						√
APL_1674				√		
APL_1721					√	
APL_1759					√	
APL_1769					√	
APL_1781					√	
APL_1782					√	
APL_1785					√	
APL_1793			√			
Natural Infection [20]						
Up-regulated						
APL_0163						√
APL_0339						√
APL_0375						√
APL_0450						√
APL_0668		√				√
APL_0815				√		
APL_0920			√			
APL_1494						√
APL_1665	√					
APL_2011				√		
APL_2025			√			
APL_2026			√			
Down-regulated						
APL_0226				√	√	
APL_0333						√
APL_0615						√
APL_0630						√
APL_0644				√		
APL_0682					√	
APL_0771		√		√		
APL_0903			√			
APL_0967			√			
APL_0982			√		√	
APL_1136						√
APL_1169					√	
APL_1292			√			
APL_1388	√		√			
APL_1450				√		
APL_1474					√	
APL_1558					√	√
APL_1597						
APL_1759					√	
APL_1782					√	
APL_1962					√	

Interestingly, APL_1494, which encodes the fine tangled pili,was down-regulated in the drip-flow biofilm and in bacteria attached to SJPL cells [19]. This gene was also up-regulated in a natural infection [21], in a growth medium favoring biofilm formation [6] and in *A. pleuropneumoniae* cultured in the presence of SJPL cells [19]. Another protein of interest identified in our analysis is the autotransporter adhesin APL_0443 which was down-regulated in the 4 h vs 6 h biofilm experiment. This adhesin is also down-regulated in the presence of epinephrine and up-regulated in the presence of norepinephrine [39]. Furthermore, *A. pleuropneumoniae* adhered more to SJPL cells when treated with norepinephrine [39]. However, the presence of epinephrine or norepinephrine did not have an effect on biofilm formation [39]. Differential regulation of APL_0443 has also been observed before; APL_0443 was up-regulated when *A. pleuropneumoniae* was cultured in a growth medium favouring biofilm formation [6] and in the presence of porcine bronchoalveolar lavage fluid [20] and was down-regulated in *A. pleuropneumoniae* attached to SJPL cells [21]. Based on the observations mentioned above, both APL_1494 and APL_0443 might play a role during early adhesion of *A. pleuropneumoniae,* more specifically the reversible attachment step of biofilm formation*.*

## Conclusion

The formation of a static biofilm in a microtiter plate by *A. pleuropneumoniae* strain S4074 is a rapid process that reaches its peak by 5 h and is fully dispersed by 7 h. Furthermore, *A. pleuropneumoniae* can form a robust biofilm under low-shear force in a drip-flow apparatus and this helps to overcome the limitation of a microtiter plate for future biofilm studies. Additionally, the dripflow biofilm reactor may represent the better model to study the formation of biofilm by *A. pleuropneumoniae*. Transcriptional analyses also indicated that the formation of a biofilm under low-shear force requires a different sub-set of genes than a biofilm grown under static conditions. Candidates involved in early attachment, and dispersion were identified for future work.

## Methods

### Bacterial strain and growth conditions

*A. pleuropneumoniae* strain S4074 was routinely cultured in brain heart infusion broth (BHI; Oxoid Ltd, Basingstoke, Hampshire, England) or on BHI agar supplemented with 5 or 15 μg/mL NAD (BHI-NAD), respectively. *A. pleuropneumoniae* on BHI-NAD agar were incubated for 24 h at 37°C with 5% CO_2_. Overnight cultures of *A. pleuropneumoniae* were incubated for 16 h at 37°C with shaking (~200 rpm).

### Biofilms under static conditions

Biofilms were cultured in 96-well microtiter plates (Costar^®^ 3599; Corning, Corning, NY, USA) as described by Labrie et al. [6] with some modifications. Briefly, an overnight culture of *A. pleuropneumoniae* strain S4074 was inoculated (1% v/v) into fresh BHI-NAD and 100 μL of this inoculum was transferred into 3 wells. The microtiter plate was incubated at 37°C with 5% CO_2_. After the desired incubation time, the liquid medium was removed using a vacuum and the planktonic cells were removed by washing the well once with MilliQ water. The biofilms were then stained with 0.1% (w/v) crystal violet for 2 min. The biofilms were washed once with distilled water and then dried at 37°C for 15 min. The stain was then released with 100 μL of 75% (v/v) ethanol and the amount of released stain was quantified by measuring the absorbance at 590 nm with a microplate reader. For RNA extraction purposes, biofilms were cultured (37°C, 5% CO_2_) in 6-well plates containing 4.5 mL culture of *A. pleuropneumoniae*. After the desired incubation time, the liquid medium containing the planktonic cells was harvested and 0.1 volume of stop solution (95% (v/v) ethanol, 5% (v/v) buffer-saturated phenol) was added. The plate was washed once with MilliQ water, and the attached cells were resuspended in PBS containing 10% (v/v) stop solution. The bacteria in the stop solution were stored at –80°C until RNA was isolated.

### Biofilms in a drip-flow apparatus

Biofilms were cultured in a drip-flow apparatus (DFR 110 Biofilm Reactor, BioSurface Technologies Corp. Bozeman Montana, USA) as described by Goeres et al. [22] with some modifications. Briefly, an overnight culture of *A. pleuropneumoniae* strain S4074 was inoculated (1% v/v) into fresh BHI-NAD and 12.5 mL of this inoculum was transferred into a channel containing a glass slide (Catalogue #48300-025, VWR, Ville Mont-Royal, Quebec, Canada). The apparatus was incubated for 4 h at 37°C with 5% CO_2_ to allow the biofilm to form under static conditions. The apparatus legs were then attached to create a 10° downward slope. The apparatus was then connected to the nutrient system containing pre-warmed (37°C) 50% BHI-NAD. The flow (~200 mL per hour per channel) of the medium was then initiated and maintained for 24 h at 37°C. After 24 h, the glass slide was removed and gently washed once with sterile MiliQ water. The biofilm was either stored at –80°C in PBS containing 10% (v/v) stop solution or used in confocal microscopy as described below. For the microarray experiments, the effluent bacteria were collected for 1h after the 23^rd^ hour of flow. To preserve the RNA, 0.1 volume of stop solution was added to the collected bacteria and stored at –80°C. These bacteria were considered to be the planktonic population.

### RNA isolation

RNA was isolated with acid phenol as described by Deslandes et al. [21] with some modifications. Briefly, bacteria in the stop solution were harvested by centrifugation (4000 × g, 30 min, 4°C) and resuspended in 600 μL of PBS. A volume (300 μL) of pre-warmed (100°C) lysis solution was added to the bacterial suspension and the resulting mixture was incubated at 100°C for 5 min. A volume (900 μL) of pre-warmed (65°C) acid phenol:chloroform was added to the lysed bacteria and this mixture was incubated at 65°C for 5 min. The aqueous phase was separated by centrifugation (14 000 × g, 10 min) and extracted twice with 1 volume of acidic phenol:chloroform (14 000 × g, 10 min). The aqueous phase was then treated twice with 1 volume of chloroform (14 000 × g, 10 min). Sodium acetate (to 0.3 M) was added to the aqueous phase and the RNA was precipitated with 1 volume of isopropyl alcohol for 20 min at -20°C. The RNA was collected by centrifugation (14 000 × g, 20 min) and washed with 75% (v/v) ethanol. The precipitated RNA was resuspended in RNase-free water (100 μL) and was treated with turbo DNase (Ambion, TX, USA) as prescribed by the manufacturer. The RNA was then precipitated and washed as described above, and the precipitated RNA was resuspended in RNase-free water (100 μL) and stored at –20˚C.

### Microarray hybridization and analysis

Microrrays hybridization and analysis were performed as described previously [18]. Briefly, cDNA was synthesized from 15 μg of RNA isolated from the reference (planktonic cells) and the experimental conditions (biofilm cells) and indirectly labelled using a monofunctional NHS-ester Cy3 or Cy5 dye (GE Healthcare, Baie d’Urfé, QC, Canada). Labelling efficiency was determined using a Nanodrop ND-1000 spectrophotometer (Nanodrop, Rockland, DE, USA) and labelled samples were combined. Samples were then added to the AppChip2 microarrays slide (for description of the design see [19] and [21]) and hybridized overnight. Images of the microarray slides were acquired using a Perkin-Elmer ScanArray Express scanner. Each comparison was performed with 4 arrays which included three biological replicates, one technical replicate and a dye swap for one of the biological replicate. Microarray images were analyzed with the TM4 Microarray Software Suite [40] as described previously [18]. Raw data was generated using Spotfinder v.3.1.1 and were normalized with the MIDAS software using cross-channel Loess normalization. Significance was set to q=0.000 with a false discovery rate (FDR) set to 0.000. Data were submitted to the Gene Expression Omnibus [41] [GEO: GSE43824].

### Quantitative real-time reverse-transcriptase PCR (qRT-PCR)

To confirm microarray results, qRT-PCR was performed as described elsewhere [21]. Briefly, 25 μl reactions were prepared in triplicate using the QuantiTect^®^ SYBR^®^ Green RT-PCR Kit (Qiagen) and amplification was performed with a 16-place Cepheid Smart Cycler^®^ System. Relative expression was normalized to *rluC* which appeared to have a constant expression throughout the different microarray experiments (data not shown). Quantitative measures were obtained using the 2^-∆∆CT^ method [42].

### Confocal laser scanning microscopy

Biofilms were prepared as described in section above but were stained with the live/dead markers (Invitrogen, Eugene, OR, USA) or wheat-germ agglutinin (WGA) conjugated with oregon green (Invitrogen) as prescribed by the manufacturer. For the drip-flow glass slide, 1 mL of the staining solution was deposited on the slide and once the staining procedure was complete, the slide was immersed in water or PBS contained in a petri dish. The stained biofilms were visualized by confocal laser scanning microscopy (Olympus FV1000 IX81, Markham, ON, Canada) and images were acquired using the Fluoview software (Olympus). To obtain the thickness and volume values, biofilm images were analyzed using the Image Pro 9.0 software (Media Cybernetics, Inc. Bethesda, MD, USA). Briefly, a 3D image of each biofilm was generated using the 15 image layers and an isoimage was created from the reconstructed 3D image. This isoimage was used to measure the volume and thickness of the biofilm. Biomass was obtained by dividing the total biofilm volume over the surface area of the field of view.

### Statistical analysis

Statistical significance for the biomass and biofilm assays was tested by analysis of variance (ANOVA; 95% confidence interval) followed by a Tukey’s post hoc comparison (*p* < 0.05) using Graph Pad version 4.02 (GraphPad Software, San Diego, CA, USA).

## Competing interests

The authors declare that they have no competing interests.

## Authors’ contribution

YDNT design the study, carried all experiments and drafted the manuscript. VD helped design the study and reviewed the manuscript. MJ conceived the study, helped with the design and reviewed the manuscript. All authors read and approved the final manuscript.

## Supplementary Material

Additional file 1: Table S1 List of primers used in the qRT-PCR validation. **Table S2**: Genes that were significantly (FDR=0) up-regulated or down-regulated in a 4 h biofilm (biofilm vs plankton). **Table S3**: Genes that were significantly (FDR=0) up-regulated or down-regulated in a 6h biofilm (biofilm vs plankton). **Table S4**: Genes that were significantly (FDR=0) up-regulated or down-regulated in a growing biofilm (4 h biofilm vs 6 h biofilm). **Table S5**: Genes that were significantly (FDR=0) up-regulated or down-regulated in a 28 h biofilm cultured in a drip-flow apparatus (biofilm vs plankton). Click here for file
