# Supplementary material for: Actinobacillus pleuropneumoniae genes expression in biofilms cultured under static conditions and in a drip-flow apparatus
Source: BMC Genomics. 2013 May 31;14:364. doi: 10.1186/1471-2164-14-364 (PMC3671958; doi:10.1186/1471-2164-14-364)
Supplement: Additional file 1: Table S1 — List of primers used in the qRT-PCR validation. Table S2: Genes that were significantly (FDR=0) up-regulated or down-regulated in a 4 h biofilm (biofilm vs plankton). Table S3: Genes that were significantly (FDR=0) up-regulated or down-regulated in a 6h biofilm (biofilm vs plankton). Table S4: Genes that were significantly (FDR=0) up-regulated or down-regulated in a growing biofilm (4 h biofilm vs 6 h biofilm). Table S5: Genes that were significantly (FDR=0) up-regulated or down-regulated in a 28 h biofilm cultured in a drip-flow apparatus (biofilm vs plankton). [file 1471-2164-14-364-S1.docx]

**Table S1. List of primers used in the qRT-PCR validation**

| Gene | Forward Primer | Reverse Primer | Condition^1^ |
| --- | --- | --- | --- |
| *dus* | AATGGCGGCTAAAGTCAATG | TTCCTGATCCCAACCTGTTC | 1, 5, 8 |
| *ribD* | TTAGACCGCTTGGCGTAGAT | TGGGGTTTTCGCTTGTTTAC | 4, 6, 8 |
| APL_0767 | TTGCTTCTGTTTGTGCTTGC | ACCGCCGCAGTAAATAACAC | 4, 6, 8 |
| APL_1387 | TTGCCCGAAATTTATGAACC | AGCGAACGGGTTAGGTTTTT | 4, 6, 8 |
| *pbpB* | CGCACTTGAACGTCGTAATG | AAGCTGCGCATATTTGCTTT | 3, 6 |
| *ccmC* | ATACGGTTCTATGGCGGTTG | AAACAACACCAAAGCCGAAG | 8 |
| APL_0590 | TTTCGTTCTCCGTTTGTGAA | TTTACTTGCCGCTTCTTGCT | 4 |
| *infA* | GCATTGAGATGCAAGGTACG | ACGTGCGCGGAAAATAATAC | 5, 7 |
| APL_1791 | GGTGGCGAAAAAGCATTAGA | AATCCGCCATTAACACGAAG | 5 |
| *macA* | GGGACAAGCGATTCGATTTA | TTTGCTCCGAATTTTCAACC | 7 |
| *rpoE* | CGGAAAATGTGGTGCTATCC | TGCATCAATCGCTTCTCTTG | 5, 7 |
| APL_0840 | AAGCGGATAAAGCAACCTCA | GGCTTGACCTGCGTTAAATC | 5, 7 |
| *ftpA* | GACTCGTCAAACGCCTGAAT | CGCACGGATAAACCATTGTA | 7 |
| APL_1690 | GTATCGGTGCGGGTATCATC | TCCGGCTCTTTGGTAATACG | 2, 4, 6 |
| APL_0627 | TCGGATCGAAAATCAAATCC | GGATGCGTTATTCGCTGACT | 2 |
| *nhaP* | TTGCCGGTTCTTTAGGTGTC | ATAGGGTCGGTCGGAGAGAT | 2 |
| *ansB* | CGATAAACCGGTGGTACTGG | GTTTGTACGGCGGTGGTACT | 2 |
| *gntR* | TCTACCGCCGTAAAATGGAG | AATCTGCGTTGTTGGTGATG | 2 |
| *maeB* | AGGTAATATCGGTGCGTTGG | AGAAACATTCCGGTGCTTTG | 1, 3 |
| APL_0926 | AGCGAGCTATCCAGAAGACG | TTTTTCCGAAATTCCAGTCG | 1, 3 |
| *iscA* | CTGTTCGGGACTGGCTTATG | GCCGCAAGAATCTTTCACAT | 1, 3 |
| *nrdB* | TTCCGAAACGATCCATTCAC | CGTCCACCGTATAGCAACCT | 1, 3 |

^1^ down in a 4h biofilm (1), up in a 4h biofilm(2), down in a 6h biofilm (3), up in 6h (4), down in a growing biofilm (5), up in a growing biofilm (6), down in a drip-flow biofilm (7), up in a drip flow biofilm (8).

**Table S2. Genes that were significantly (FDR=0) up-regulated or down-regulated in a 4h biofilm (biofilm vs plankton).**

| **Locus tag** | **Gene** | **Description** | **Fold change** |
| --- | --- | --- | --- |
| APL_1690 |  | inner membrane protein | 1.79 |
| APL_1902 | *yrhG* | Hypothetical transport protein yrhG (formate/nitrite transporter) | 1.75 |
| APL_1099 |  | hypothetical protein | 1.71 |
| APL_0901 |  | ribosome-associated inhibitor A | 1.71 |
| APL_0322 | *nhaB* | Na(+)/H(+) antiporter 2 | 1.58 |
| APL_0135 | *ansB* | L-asparaginase periplasmic precursor | 1.55 |
| APL_0689 | *torY* | cytochrome c-type protein TorY | 1.53 |
| APL_1532 | *dcuB* | anaerobic C4-dicarboxylate transporter DcuB | 1.51 |
| APL_1388 |  | ABC transporter permease | 1.48 |
| APL_1856 |  | hypothetical protein | 1.48 |
| APL_0674 |  | membrane protein | 1.48 |
| APL_0571 | *gntR* | HTH-type transcriptional regulator | 1.45 |
| APL_0456 | *znuC* | high-affinity zinc uptake system ATP-binding protein | 1.45 |
| APL_0372 | *corC* | magnesium and cobalt efflux protein | 1.42 |
| APL_0797 | *tyrR* | transcriptional regulatory protein TyrR | 1.41 |
| APL_1449 | *mpl* | UDP-N-acetylmuramate:L-alanyl-gamma-D-glutamyl-meso-diaminopimelate ligase | 1.39 |
| APL_0627 | *cpxA* | CpxA | 1.37 |
| APL_1004 | *nhaP* | Na(+)/H(+) exchanger beta-like protein | 1.36 |
| APL_1373 |  | DNA replication initiation factor | 1.35 |
| APL_0121 |  | hypothetical protein | 1.35 |
| APL_0837 |  | hypothetical protein | 1.34 |
| APL_1835 | *glpT* | Glycerol-3-phosphate transporter | 1.31 |
| APL_0926 |  | hypothetical protein | -1.28 |
| APL_1760 | *rplC* | 50S ribosomal protein L3 | -1.29 |
| APL_0929 | *iscA* | iron-binding protein | -1.32 |
| APL_1761 | *rplD* | 50S ribosomal protein L4 | -1.35 |
| APL_1898 |  | lipoprotein | -1.36 |
| APL_0381 | *glpC* | anaerobic glycerol-3-phosphate dehydrogenase subunit C | -1.37 |
| APL_0930 | *nifU* | scaffold protein | -1.39 |
| APL_0486 | *maeB* | NADP-dependent malic enzyme | -1.39 |
| APL_1282 | *dnaQ* | DNA polymerase III subunit epsilon | -1.4 |
| APL_1198 |  | NAD(P)H nitroreductase | -1.43 |
| APL_0636 | *hsdR3* | type I site-specific restriction-modification system, restriction subunit | -1.43 |
| APL_0993 | *nrdB* | ribonucleotide-diphosphate reductase subunit beta | -1.43 |
| APL_1762 | *rplW* | 50S ribosomal protein L23 | -1.47 |
| APL_1736 | *hslV* | ATP-dependent protease | -1.47 |
| APL_0106 | *putA* | bifunctional proline dehydrogenase/pyrroline-5-carboxylate dehydrogenase | -1.5 |
| APL_0189 | *dus* | tRNA-dihydrouridine synthase B | -1.51 |
| APL_1665 | *gntP_1* | gluconate permease | -1.58 |
| APL_1420 | *mglB* | D-galactose-binding periplasmic protein precursor | -1.6 |
| APL_0992 | *nrdA* | ribonucleoside-diphosphate reductase alpha subunit | -1.61 |
| APL_0379 | *glpA* | anaerobic glycerol-3-phosphate dehydrogenase subunit A | -1.67 |
| APL_0894 | *fdxH* | formate dehydrogenase, iron-sulfur subunit | -1.69 |
| APL_0668 |  | hypothetical protein | -1.69 |
| APL_0771 |  | dihydrolipoamide dehydrogenase | -1.96 |
| APL_0895 | *fdnI* | formate dehydrogenase, cytochrome b556 subunit | -1.98 |

**Table S3. Genes that were significantly (FDR=0) up-regulated or down-regulated in a 6h biofilm (biofilm vs plankton).**

| **Locus tag** | **Gene** | **Description** | **Fold change** |
| --- | --- | --- | --- |
| APL_1691 |  | ABC transporter, permease protein, amino acid | 2.53 |
| APL_1373 |  | DNA replication initiation factor | 2.27 |
| APL_0767 | *sst* | serine/threonine transporter | 2.01 |
| APL_0715 |  | ABC transport system permease protein | 1.98 |
| APL_0585 |  | hypothetical protein (AcrR family) | 1.97 |
| APL_0299 |  | hypothetical protein (Predicted thioesterase) | 1.97 |
| APL_1690 |  | inner membrane protein | 1.96 |
| APL_1582 | *cpxD* | capsule polysaccharide export protein | 1.94 |
| APL_1897 | *nudH* | dinucleoside polyphosphate hydrolase | 1.78 |
| APL_0982 | *rpmE* | 50S ribosomal protein L31 | 1.78 |
| APL_0006 | *ompP2A* | outer membrane protein P2 | 1.76 |
| APL_1554 | *wecA* | undecaprenyl-phosphate alpha-N-acetylglucosaminyl 1-phosphate transferase | 1.73 |
| APL_1037 | *focA* | formate transporter | 1.71 |
| APL_1385 | *plsX* | glycerol-3-phosphate acyltransferase | 1.69 |
| APL_0321 | *dsbB* | disulfide bond formation protein B | 1.69 |
| APL_0300 | *tolQ* | colicin transport protein | 1.68 |
| APL_0363 |  | hypothetical protein (coproporphyrinogen III oxidase) | 1.66 |
| APL_1292 |  | ABC transporter ATP-binding protein | 1.65 |
| APL_0382 | *ribD* | riboflavin biosynthesis protein | 1.64 |
| APL_0575 | *deaD* | cold-shock DEAD box protein A-like protein | 1.61 |
| APL_1926 | *truD* | tRNA pseudouridine synthase D | 1.58 |
| APL_1387 |  | hypothetical protein (metal-binding/nucleic acid-binding protein) | 1.55 |
| APL_0967 | *gltS* | sodium/glutamate symport carrier protein | 1.55 |
| APL_0118 | *cspC* | cold shock-like protein | 1.54 |
| APL_0920 |  | hypothetical protein (Periplasmic protein) | 1.52 |
| APL_1291 |  | hypothetical protein (permease) | 1.51 |
| APL_1228 | *infA* | translation initiation factor IF-1 | 1.49 |
| APL_2025 | *hisH* | imidazole glycerol phosphate synthase subunit | 1.47 |
| APL_1793 |  | Fe(III) dicitrate ABC transporter, ATP-binding protein | 1.46 |
| APL_1388 |  | ABC transporter permease | 1.46 |
| APL_1655 | *gidA* | tRNA uridine 5-carboxymethylaminomethyl modification enzyme | 1.45 |
| APL_1570 | *exbB* | biopolymer transport ExbB protein | 1.45 |
| APL_1896 |  | hypothetical protein (permeases) | 1.43 |
| APL_1213 |  | hypothetical protein (Predicted phosphatase/phosphohexomutase) | 1.43 |
| APL_1653 |  | F0F1 ATP synthase subunit I | 1.43 |
| APL_1901 | *rraA* | ribonuclease activity regulator protein | 1.42 |
| APL_0690 | *moaA* | molybdenum cofactor biosynthesis protein A | 1.41 |
| APL_0373 |  | hypothetical protein | 1.39 |
| APL_0456 | *znuC* | high-affinity zinc uptake system ATP-binding protein | 1.39 |
| APL_2026 | *hisA* | 1-(5-phosphoribosyl)-5-[(5-phosphoribosylamino)methylideneamino]imidazole-4-carboxamide isomerase | 1.38 |
| APL_0903 | *folE* | GTP cyclohydrolase I | 1.37 |
| APL_0677 |  | hypothetical protein | 1.37 |
| APL_0146 | *dusA* | tRNA-dihydrouridine synthase A | 1.36 |
| APL_0943 |  | N5-glutamine S-adenosyl-L-methionine-dependent methyltransferase | 1.36 |
| APL_0579 | *mukF* | condensin subunit F | 1.35 |
| APL_0716 |  | iron(III) ABC transporter, permease protein | 1.35 |
| APL_1791 |  | periplasmic iron/siderophore binding protein | 1.34 |
| APL_1461 | *menA* | 1,4-dihydroxy-2-naphthoate octaprenyltransferase | 1.33 |
| APL_0590 |  | hypothetical protein (Exopolysaccharide biosynthesis protein) | 1.3 |
| APL_0661 | *purK* | phosphoribosylaminoimidazole carboxylase ATPase subunit | -1.27 |
| APL_0764 |  | hypothetical protein | -1.27 |
| APL_1676 | *dmsC* | anaerobic dimethyl sulfoxide reductase chain C | -1.28 |
| APL_0739 | *ihfB* | integration host factor subunit beta | -1.29 |
| APL_1027 |  | hypothetical protein (Permeases of the major facilitator superfamily) | -1.29 |
| APL_0726 |  | carbonic anhydrase | -1.29 |
| APL_1537 | *prmA* | ribosomal protein L11 methyltransferase | -1.3 |
| APL_1125 |  | cysteine desulfurase | -1.3 |
| APL_0993 | *nrdB* | ribonucleotide-diphosphate reductase subunit beta | -1.3 |
| APL_0815 |  | hypothetical protein | -1.31 |
| APL_1252 |  | hypothetical protein (Di- and tricarboxylate transporters) | -1.31 |
| APL_1641 |  | hypothetical protein | -1.31 |
| APL_1455 | *ilvA* | threonine dehydratase | -1.31 |
| APL_1436 | *rne* | ribonuclease E | -1.32 |
| APL_1988 | *hemB* | delta-aminolevulinic acid dehydratase | -1.32 |
| APL_1510 | *gpsA* | NAD(P)H-dependent glycerol-3-phosphate dehydrogenase | -1.33 |
| APL_0824 | *purB* | adenylosuccinate lyase | -1.33 |
| APL_0926 |  | hypothetical protein | -1.33 |
| APL_1478 | *vacB* | ribonuclease R | -1.34 |
| APL_0249 | *thrB* | homoserine kinase | -1.35 |
| APL_1675 | *dmsB* | anaerobic dimethyl sulfoxide reductase chain B | -1.35 |
| APL_1659 |  | hypothetical protein | -1.35 |
| APL_0644 | *pta* | phosphate acetyltransferase | -1.36 |
| APL_0717 |  | iron(III) ABC transporter, ATP-binding protein | -1.36 |
| APL_0940 |  | hypothetical protein (dithiobiotin synthetase) | -1.37 |
| APL_0925 | *hscA* | chaperone protein HscA | -1.39 |
| APL_0971 |  | acyl CoA thioester hydrolase | -1.39 |
| APL_0226 |  | hypothetical protein (RecA-superfamily ATPases implicated in signal transduction) | -1.4 |
| APL_1634 |  | glycosyltransferase | -1.41 |
| APL_0941 | *bioF* | 8-amino-7-oxononanoate synthase | -1.41 |
| APL_0823 | *glpR* | glycerol-3-phosphate regulon repressor | -1.42 |
| APL_0889 |  | hypothetical protein | -1.42 |
| APL_0486 | *maeB* | NADP-dependent malic enzyme | -1.42 |
| APL_1538 |  | acetyltransferase | -1.43 |
| APL_0930 | *nifU* | scaffold protein | -1.43 |
| APL_1823 | *pbpB* | penicillin-binding protein 1B (PBP1b) | -1.43 |
| APL_0737 |  | tetratricopeptide repeat protein | -1.45 |
| APL_1547 | *hcr* | HCP oxidoreductase, NADH-dependent | -1.46 |
| APL_0928 | *hscB* | co-chaperone HscB | -1.47 |
| APL_1946 |  | hypothetical protein (membrane protein) | -1.48 |
| APL_1674 | *dmsA* | anaerobic dimethyl sulfoxide reductase chain A precursor | -1.48 |
| APL_1282 | *dnaQ* | DNA polymerase III subunit epsilon | -1.49 |
| APL_0888 | *fadJ* | putative fatty acid oxidation complex alpha subunit | -1.52 |
| APL_1415 |  | hypothetical protein | -1.54 |
| APL_0740 | *rpsA* | 30S ribosomal protein S1 | -1.55 |
| APL_0921 |  | hypothetical protein (TPR repeat) | -1.56 |
| APL_0383 | *ribE* | riboflavin synthase subunit alpha | -1.62 |
| APL_1959 | *adhI* | alcohol dehydrogenase | -1.64 |
| APL_0444 |  | hypothetical protein | -1.67 |
| APL_0445 | *ykgF* | electron transport protein | -1.69 |
| APL_2010 | *hbpA* | Heme-binding protein A | -1.7 |
| APL_1765 | *rplV* | 50S ribosomal protein L22 | -1.71 |
| APL_0896 | *fdhE* | formate dehydrogenase accessory protein FdhE | -1.75 |
| APL_0384 | *ribA* | riboflavin biosynthesis protein | -1.83 |
| APL_0929 | *iscA* | iron-binding protein | -1.83 |
| APL_1414 | *mqo* | malate:quinone oxidoreductase | -2.17 |
| APL_0892 | *fdxG* | formate dehydrogenase, nitrate-inducible, major subunit | -2.24 |
| APL_0106 | *putA* | bifunctional proline dehydrogenase/pyrroline-5-carboxylate dehydrogenase | -2.45 |
| APL_0107 | *putP* | sodium/proline symporter | -2.46 |
| APL_0687 | *dld* | D-lactate dehydrogenase | -2.61 |
| APL_0894 | *fdxH* | formate dehydrogenase, iron-sulfur subunit | -2.73 |
| APL_0771 | *lpdA* | dihydrolipoamide dehydrogenase | -2.78 |
| APL_0893 | *fdxG* | formate dehydrogenase, nitrate-inducible, major subunit | -2.79 |
| APL_2011 | *aldA* | putative aldehyde dehydrogenase | -3.01 |
| APL_0895 | *fdnI* | formate dehydrogenase, cytochrome b556 subunit | -3.06 |
| APL_1977 | *yedE* | inner membrane protein | -3.09 |
| APL_1976 | *yedF* | hypothetical protein (redox protein, regulator of disulfide bond formation) | -3.32 |
| APL_1450 | *fbp* | fructose-1,6-bisphosphatase | -3.57 |

**Table S4. Genes that were significantly (FDR=0) up-regulated or down-regulated in a growing biofilm (4h biofilm vs 6h biofilm).**

| **Locus tag** | **Gene** | **Description** | **Fold change** |
| --- | --- | --- | --- |
| APL_1676 | *dmsC* | anaerobic dimethyl sulfoxide reductase chain C | 10.53 |
| APL_1678 | *napF* | ferredoxin | 7.84 |
| APL_1677 | *dmsD* | twin-argninine leader-binding protein | 7.48 |
| APL_0343 | *fruA* | PTS system fructose-specific EIIBC component | 7.13 |
| APL_1675 | *dmsB* | anaerobic dimethyl sulfoxide reductase chain B | 6.94 |
| APL_0345 | *fruB* | multiphosphoryl transfer protein | 6.1 |
| APL_0740 | *rpsA* | 30S ribosomal protein S1 | 5.87 |
| APL_2010 | *hbpA* | Heme-binding protein A | 5.8 |
| APL_0153 | *nqrD* | Na(+)-translocating NADH-quinone reductase subunit D | 5.72 |
| APL_1209 | *betT* | choline-glycine betaine transporter | 5.66 |
| APL_1252 |  | hypothetical protein (Di- and tricarboxylate transporters) | 4.73 |
| APL_0767 | *sst* | serine/threonine transporter | 4.69 |
| APL_0842 | *pntA* | NAD(P) transhydrogenase subunit alpha | 4.64 |
| APL_0841 | *pntB* | NAD(P) transhydrogenase subunit beta | 4.34 |
| APL_0102 | *nrfC* | nitrate reductase | 4.08 |
| APL_0893 | *fdxG* | formate dehydrogenase, nitrate-inducible, major subunit | 4.07 |
| APL_0097 | *ilvD* | Dihydroxy-acid dehydratase | 4.04 |
| APL_1373 |  | DNA replication initiation factor | 3.95 |
| APL_0976 |  | membrane protein | 3.66 |
| APL_0895 | *fdnI* | formate dehydrogenase, cytochrome b556 subunit | 3.65 |
| APL_0967 | *gltS* | sodium/glutamate symport carrier protein | 3.63 |
| APL_0892 | *fdxG* | formate dehydrogenase, nitrate-inducible, major subunit | 3.54 |
| APL_1838 | *asnC* | Regulatory protein asnC | 3.42 |
| APL_0250 | *thrA* | bifunctional aspartokinase/homoserine dehydrogenase | 3.42 |
| APL_1787 | *rimM* | 16S rRNA-processing protein rimM | 3.41 |
| APL_1054 | *cyaA* | adenylate cyclase | 3.4 |
| APL_1769 | *rpsQ* | 30S ribosomal protein S17 | 3.34 |
| APL_0575 | *deaD* | cold-shock DEAD box protein A-like protein | 3.29 |
| APL_1660 |  | mannose permease IID component | 3.28 |
| APL_1370 | *ccmC* | heme exporter protein C | 3.28 |
| APL_1387 |  | hypothetical protein (metal-binding/nucleic acid-binding protein) | 3.25 |
| APL_1392 | *ptnC* | mannose permease IIC component | 3.24 |
| APL_1760 | *rplC* | 50S ribosomal protein L3 | 3.22 |
| APL_0527 |  | ABC-type transport system, permease component | 3.2 |
| APL_0382 | *ribD* | riboflavin biosynthesis protein | 3.2 |
| APL_0320 | *metC* | cystathionine beta-lyase | 3.15 |
| APL_0593 | *guaB* | inosine-5-monophosphate dehydrogenase | 3.11 |
| APL_0349 | *glgA* | glycogen synthase | 3.11 |
| APL_1762 | *rplW* | 50S ribosomal protein L23 | 3.09 |
| APL_0255 | *gpt* | xanthine phosphoribosyltransferase | 3.07 |
| APL_1789 | *rplS* | 50S ribosomal protein L19 | 3.07 |
| APL_1319 | *ptsB* | PTS system sucrose-specific EIIBC component | 3.06 |
| APL_1316 | *dcuB2* | anaerobic C4-dicarboxylate transporter | 3.04 |
| APL_0718 |  | hypothetical protein | 3.04 |
| APL_1695 | *kpsC* | capsule polysaccharide modification protein LipA | 3.03 |
| APL_1781 | *rpsM* | 30S ribosomal protein S13 | 3.02 |
| APL_1759 | *rpsJ* | 30S ribosomal protein S10 | 3 |
| APL_1962 | *hflX* | GTP-binding protein hflX | 2.99 |
| APL_1149 | *aspS* | aspartyl-tRNA synthetase | 2.99 |
| APL_1204 |  | esterase | 2.98 |
| APL_0735 |  | hypothetical protein | 2.97 |
| APL_1765 | *rplV* | 50S ribosomal protein L22 | 2.96 |
| APL_0804 |  | hypothetical protein | 2.96 |
| APL_1647 | *atpG* | ATP synthase gamma chain | 2.93 |
| APL_0587 | *acrB* | acriflavine resistance protein | 2.93 |
| APL_0256 | *gmk* | guanylate kinase | 2.91 |
| APL_0847 |  | oxygen-independent coproporphyrinogen III oxidase-like protein | 2.91 |
| APL_1529 | *frdA* | fumarate reductase flavoprotein subunit | 2.9 |
| APL_0894 | *fdxH* | formate dehydrogenase, iron-sulfur subunit | 2.9 |
| APL_1766 | *rpsC* | 30S ribosomal protein S3 | 2.88 |
| APL_0977 | *xthA* | exodeoxyribonuclease III | 2.87 |
| APL_1447 | *afuB* | Ferric transport system permease protein fbpB | 2.86 |
| APL_1553 |  | hypothetical protein | 2.85 |
| APL_1385 | *plsX* | glycerol-3-phosphate acyltransferase | 2.83 |
| APL_0195 | *mrcA* | membrane carboxypeptidase/penicillin-binding protein | 2.82 |
| APL_1372 | *ccmA* | cytochrome c biogenesis ATP-binding export protein | 2.8 |
| APL_0983 | *tktA* | transketolase 2 | 2.77 |
| APL_0644 | *pta* | phosphate acetyltransferase | 2.74 |
| APL_1171 | *rpsF* | 30S ribosomal protein S6 | 2.74 |
| APL_1763 | *rplB* | 50S ribosomal protein L2 | 2.73 |
| APL_0100 | *nrfA* | Cytochrome c-552 precursor | 2.73 |
| APL_1783 | *rpsD* | 30S ribosomal protein S4 | 2.72 |
| APL_0700 | *hisC* | histidinol-phosphate aminotransferase 2 | 2.72 |
| APL_0414 | *uppS* | undecaprenyl pyrophosphate synthetase | 2.71 |
| APL_1268 |  | protease 4 | 2.71 |
| APL_1947 | *nhaA* | Na(+)/H(+) antiporter 1 (Sodium/proton antiporter 1) | 2.7 |
| APL_0344 | *fruK* | 1-phosphofructokinase | 2.7 |
| APL_1646 | *atpD* | ATP synthase subunit beta | 2.7 |
| APL_1310 | *pgl* | 6-phosphogluconolactonase | 2.67 |
| APL_1772 | *rpsN* | 30S ribosomal protein S14 | 2.67 |
| APL_1181 | *kefB* | glutathione-regulated potassium-efflux system protein | 2.66 |
| APL_1662 |  | PTS system mannose-specific EIIAB component | 2.66 |
| APL_1995 | *ispB* | octaprenyl-diphosphate synthase | 2.65 |
| APL_0595 | *ftsH* | cell division protease FtsH-like protein | 2.65 |
| APL_1865 | *accC* | Biotin carboxylase | 2.64 |
| APL_1897 | *nudH* | dinucleoside polyphosphate hydrolase | 2.63 |
| APL_1312 | *cysQ* | CysQ-like protein | 2.62 |
| APL_1016 |  | nucleoside transporter | 2.61 |
| APL_1191 | *namA* | NADPH dehydrogenase | 2.61 |
| APL_1079 |  | purine permease | 2.59 |
| APL_1053 | *kefBC* | glutathione-regulated potassium-efflux system protein | 2.59 |
| APL_0449 |  | integral membrane protein | 2.57 |
| APL_0778 | *msbA* | lipid A export ATP-binding/permease protein MsbA | 2.56 |
| APL_1105 | *dnaE* | DNA polymerase III subunit alpha | 2.55 |
| APL_1912 | *xylR* | xylose operon regulatory protein | 2.55 |
| APL_1649 | *atpH* | ATP synthase delta chain | 2.54 |
| APL_1291 |  | hypothetical protein (permease) | 2.54 |
| APL_1824 | *menB* | naphthoate synthase | 2.53 |
| APL_0775 | *prsA* | ribose-phosphate pyrophosphokinase | 2.51 |
| APL_1010 | *hemC* | porphobilinogen deaminase | 2.51 |
| APL_1843 | *cysJ* | Sulfite reductase [NADPH] flavoprotein alpha-component (SIR-FP) | 2.5 |
| APL_0211 | *glyA* | glycine/serine hydroxymethyltransferase | 2.49 |
| APL_1993 | *fabD* | Malonyl CoA-acyl carrier protein transacylase (MCT) | 2.49 |
| APL_0383 | *ribE* | riboflavin synthase subunit alpha | 2.47 |
| APL_0675 | *asnS* | asparaginyl-tRNA synthetase | 2.46 |
| APL_0173 |  | hypothetical protein | 2.46 |
| APL_1902 | *yrhG* | Hypothetical transport protein yrhG | 2.45 |
| APL_1650 | *atpF* | ATP synthase B chain | 2.45 |
| APL_1552 | *wecB* | UDP-N-acetylglucosamine 2-epimerase | 2.44 |
| APL_1652 | *atpB* | ATP synthase A chain | 2.43 |
| APL_0887 | *fadI* | 3-ketoacyl-CoA thiolase | 2.42 |
| APL_0777 | *lolB* | outer-membrane lipoprotein LolB precursor | 2.42 |
| APL_0346 | *glgB* | 1,4-alpha-glucan branching enzyme | 2.41 |
| APL_1847 | *cysW* | Sulfate transport system permease protein | 2.4 |
| APL_1309 |  | permease | 2.4 |
| APL_1248 |  | AzlC family protein | 2.39 |
| APL_0375 | *glpK* | glycerol kinase | 2.39 |
| APL_1043 |  | aminotransferase | 2.39 |
| APL_0992 | *nrdA* | ribonucleoside-diphosphate reductase alpha subunit | 2.37 |
| APL_0431 |  | hypothetical protein | 2.35 |
| APL_0690 | *moaA* | molybdenum cofactor biosynthesis protein A | 2.34 |
| APL_0099 | *ilvG* | Acetolactate synthase isozyme II large subunit (AHAS-II) | 2.33 |
| APL_1782 | *rpsK* | 30S ribosomal protein S11 | 2.32 |
| APL_1785 | *rplQ* | 50S ribosomal protein L17 | 2.31 |
| APL_1376 | *oadA* | oxaloacetate decarboxylase alpha chain | 2.3 |
| APL_0727 | *ilvI* | acetolactate synthase large subunit | 2.3 |
| APL_0973 |  | membrane protein | 2.3 |
| APL_1851 | *murB* | UDP-N-acetylenolpyruvoylglucosamine reductase | 2.3 |
| APL_1882 |  | hypothetical protein | 2.29 |
| APL_1371 | *ccmB* | heme exporter protein B | 2.29 |
| APL_1918 | *vacJ* | lipoprotein VacJ-like protein precursor | 2.28 |
| APL_0377 | *glpT* | glycerol-3-phosphate transporter | 2.27 |
| APL_0738 |  | hypothetical protein | 2.27 |
| APL_0966 |  | transport protein | 2.27 |
| APL_1839 | *udp* | uridine phosphorylase | 2.26 |
| APL_1784 | *rpoA* | DNA-directed RNA polymerase alpha chain | 2.25 |
| APL_0660 | *ansB* | L-asparaginase | 2.24 |
| APL_0452 | *sucC* | succinyl-CoA synthetase beta chain | 2.24 |
| APL_1384 | *fabH* | 3-oxoacyl-[acyl-carrier-protein] synthase 3 | 2.24 |
| APL_1554 | *wecA* | undecaprenyl-phosphate alpha-N-acetylglucosaminyl 1-phosphate transferase | 2.24 |
| APL_1588 |  | TRAP transporter solute receptor | 2.23 |
| APL_1222 |  | aspartate-semialdehyde dehydrogenase | 2.23 |
| APL_1205 |  | glutathione S-transferase | 2.21 |
| APL_0769 | *ushA* | UshA precursor | 2.21 |
| APL_0249 | *thrB* | homoserine kinase | 2.21 |
| APL_1448 | *afuC* | ferric ABC transporter ATP-binding protein | 2.2 |
| APL_0826 | *cydC* | transport ATP-binding protein CydC | 2.2 |
| APL_0298 | *cydB* | cytochrome oxidase subunit 2 | 2.18 |
| APL_0136 | *pyrG* | CTP synthase | 2.17 |
| APL_1713 |  | membrane protein | 2.16 |
| APL_1550 | *wecD* | putative TDP-D-fucosamine acetyltransferase | 2.16 |
| APL_0661 | *purK* | phosphoribosylaminoimidazole carboxylase ATPase subunit | 2.16 |
| APL_1073 |  | hypothetical protein | 2.15 |
| APL_0594 | *ftsJ* | ribosomal RNA large subunit methyltransferase J | 2.15 |
| APL_2016 |  | Ferrioxamine B receptor precursor | 2.15 |
| APL_1478 | *vacB* | ribonuclease R | 2.15 |
| APL_1582 | *cpxD* | capsule polysaccharide export protein | 2.14 |
| APL_0771 | *lpdA* | dihydrolipoamide dehydrogenase | 2.13 |
| APL_0732 | *ubiX* | 3-octaprenyl-4-hydroxybenzoate carboxy-lyase | 2.13 |
| APL_0568 |  | hypothetical protein | 2.12 |
| APL_0145 |  | hypothetical protein | 2.12 |
| APL_1823 | *pbpB* | penicillin-binding protein 1B (PBP1b) | 2.12 |
| APL_0699 | *aroA* | 3-phosphoshikimate 1-carboxyvinyltransferase | 2.11 |
| APL_1934 |  | hypothetical protein | 2.11 |
| APL_1896 |  | hypothetical protein (permeases) | 2.1 |
| APL_1359 | *dsbD* | thiol:disulfide interchange protein DsbD precursor | 2.1 |
| APL_1208 | *adhC* | alcohol dehydrogenase class 3 | 2.1 |
| APL_0972 | *ispZ* | intracellular septation protein | 2.09 |
| APL_1243 | *gor* | glutathione reductase | 2.09 |
| APL_1984 | *ubiB* | ubiquinone biosynthesis protein ubiB | 2.09 |
| APL_1770 | *rplX* | 50S ribosomal protein L24 | 2.08 |
| APL_0933 | *ompP1* | outer membrane protein precursor | 2.08 |
| APL_0606 |  | symporter | 2.08 |
| APL_0138 | *leuD* | 3-isopropylmalate dehydratase small subunit | 2.07 |
| APL_1684 | *fucI* | L-fucose isomerase | 2.07 |
| APL_0888 | *fadJ* | putative fatty acid oxidation complex alpha subunit | 2.07 |
| APL_0578 | *parC* | DNA topoisomerase 4 subunit A | 2.07 |
| APL_0106 | *putA* | bifunctional proline dehydrogenase/pyrroline-5-carboxylate dehydrogenase | 2.06 |
| APL_1983 |  | hypothetical protein | 2.06 |
| APL_0579 | *mukF* | condensin subunit F | 2.06 |
| APL_1721 | *rplL* | 50S ribosomal protein L7/L12 | 2.05 |
| APL_0991 |  | hypothetical protein | 2.03 |
| APL_0821 | *gyrB* | DNA gyrase subunit B | 2.02 |
| APL_1632 |  | transcriptional regulator of sugar metabolism | 2.02 |
| APL_0943 |  | N5-glutamine S-adenosyl-L-methionine-dependent methyltransferase | 2.01 |
| APL_0935 | *arcB* | aerobic respiration control sensor protein | 2.01 |
| APL_0742 | *degS* | protease DegS precursor | 2.01 |
| APL_1389 |  | hypothetical protein | 2 |
| APL_1692 |  | ABC transporter, permease protein, amino acid | 2 |
| APL_1422 |  | hypothetical protein | 2 |
| APL_1895 | *lgt* | Prolipoprotein diacylglyceryl transferase | 1.99 |
| APL_1028 |  | glycosyltransferase | 1.98 |
| APL_1693 |  | amino-acid ABC transporter ATP-binding protein | 1.98 |
| APL_0747 | *mepA* | penicillin-insensitive murein endopeptidase precursor | 1.98 |
| APL_1835 | *glpT* | Glycerol-3-phosphate transporter | 1.98 |
| APL_0183 |  | Mn2+ and Fe2+ transporters of the NRAMP family | 1.98 |
| APL_0763 | *perM* | permease perM-like protein | 1.98 |
| APL_1690 |  | inner membrane protein | 1.96 |
| APL_0067 | *dppD* | dipeptide transport ATP-binding protein DppD | 1.96 |
| APL_0592 | *guaA* | GMP synthase (glutamine-hydrolyzing) | 1.95 |
| APL_1313 |  | ADP compounds hydrolase | 1.95 |
| APL_1066 | *yajC* | preprotein translocase subunit YajC | 1.95 |
| APL_1326 | *uvrD* | DNA helicase II | 1.95 |
| APL_1428 | *napG* | ferredoxin-type protein napG-like protein | 1.95 |
| APL_1532 | *dcuB* | anaerobic C4-dicarboxylate transporter DcuB | 1.94 |
| APL_1367 | *ccmF* | cytochrome c-type biogenesis protein | 1.94 |
| APL_0659 | *purE* | phosphoribosylaminoimidazole carboxylase catalytic subunit | 1.93 |
| APL_0066 | *dppC* | dipeptide transport system permease protein DppC | 1.93 |
| APL_0424 | *cvpA* | colicin V production protein | 1.93 |
| APL_2019 | *hisG* | ATP phosphoribosyltransferase (ATP-PRTase) (ATP-PRT) | 1.93 |
| APL_0432 | *leuB* | 3-isopropylmalate dehydrogenase | 1.93 |
| APL_1253 |  | putative sodium/sulphate transporter | 1.93 |
| APL_1275 | *gltX* | glutamyl-tRNA synthetase | 1.93 |
| APL_1104 |  | membrane protein | 1.91 |
| APL_0483 |  | nitroreductase | 1.91 |
| APL_1982 | *ubiE* | Ubiquinone/menaquinone biosynthesis methyltransferase | 1.89 |
| APL_1304 | *cysZ* | sulfate transport protein CysZ | 1.87 |
| APL_0305 |  | membrane protein | 1.86 |
| APL_0422 | *argG* | argininosuccinate synthase | 1.86 |
| APL_1427 | *napH* | ferredoxin-type protein NapH-like protein | 1.86 |
| APL_0708 | *ppx* | exopolyphosphatase | 1.85 |
| APL_0322 | *nhaB* | Na(+)/H(+) antiporter 2 | 1.85 |
| APL_1776 | *rpsE* | 30S ribosomal protein S5 | 1.84 |
| APL_1249 | *sapF* | peptide transport system ATP-binding protein SapF | 1.84 |
| APL_1749 |  | Putative acyl-CoA thioester hydrolase | 1.83 |
| APL_0980 | *lbgA* | LPS biosynthesis protein | 1.82 |
| APL_1540 | *tldD* | TldD-like protein | 1.82 |
| APL_0159 | *hcaT* | 3-phenylpropionic acid transporter | 1.82 |
| APL_0811 |  | hypothetical protein | 1.8 |
| APL_1673 | *rbsK* | ribokinase | 1.8 |
| APL_1198 |  | NAD(P)H nitroreductase | 1.8 |
| APL_1282 | *dnaQ* | DNA polymerase III subunit epsilon | 1.79 |
| APL_0413 | *cdsA* | phosphatidate cytidylyltransferase | 1.79 |
| APL_1042 | *menF* | menaquinone-specific isochorismate synthase | 1.78 |
| APL_0455 | *sucA* | 2-oxoglutarate dehydrogenase E1 component | 1.77 |
| APL_0710 | *lysC* | lysine-sensitive aspartokinase 3 | 1.72 |
| APL_1518 | *glnB* | nitrogen regulatory protein P-II | -1.74 |
| APL_1937 | *hemH* | Ferrochelatase | -1.75 |
| APL_0199 |  | hypothetical protein | -1.77 |
| APL_0358 | *nfo* | putative endonuclease 4 | -1.79 |
| APL_0703 |  | ATP-dependent helicase | -1.8 |
| APL_1361 |  | hypothetical protein | -1.82 |
| APL_1924 | *pgaD* | biofilm PGA synthesis protein pgaD | -1.85 |
| APL_0177 | *folK* | 2-amino-4-hydroxy-6-hydroxymethyldihydropteridine pyrophosphokinase | -1.85 |
| APL_0189 | *dus* | tRNA-dihydrouridine synthase B | -1.86 |
| APL_0859 |  | tryptophan biosynthesis protein trpCF | -1.86 |
| APL_0865 | *aape0934* | hypothetical protein | -1.86 |
| APL_1903 |  | hypothetical protein | -1.86 |
| APL_1965 | *crp* | Catabolite gene activator (cAMP receptor protein) (cAMP-regulatory protein) | -1.87 |
| APL_0614 | *bioD1* | dethiobiotin synthetase 1 | -1.87 |
| APL_0729 | *pmbA* | antibiotic maturation factor | -1.87 |
| APL_1738 |  | lactoylglutathione lyase and related lyases | -1.87 |
| APL_0075 | *ybaB* | hypothetical protein | -1.88 |
| APL_1189 |  | hypothetical protein | -1.88 |
| APL_0263 |  | putative ABC transport system permease | -1.89 |
| APL_1923 | *pgaC* | biofilm PGA synthesis N-glycosyltransferase PgaC | -1.9 |
| APL_0891 | *fdhD* | formate dehydrogenase accessory protein-like protein | -1.91 |
| APL_1568 | *tbpB* | transferrin-binding protein | -1.91 |
| APL_1360 |  | hypothetical protein | -1.92 |
| APL_0247 | *rho* | transcription termination factor Rho | -1.92 |
| APL_1605 |  | hypothetical protein | -1.93 |
| APL_0120 | *prc* | tail-specific protease precursor | -1.93 |
| APL_0364 | *ssa1 /aasP* | serotype-specific antigen 1 precursor | -1.93 |
| APL_0351 | *ndk* | nucleoside diphosphate kinase | -1.95 |
| APL_1920 |  | site-specific recombinase | -1.95 |
| APL_0040 | *yhbZ* | GTP-binding protein | -1.95 |
| APL_1513 | *coaA* | pantothenate kinase | -1.96 |
| APL_0362 | *ung* | uracil-DNA glycosylase | -1.96 |
| APL_0625 |  | hypothetical protein | -1.96 |
| APL_0626 | *macB* | macrolide-specific ABC-type efflux carrier | -1.97 |
| APL_0997 | *lacZ* | beta-galactosidase | -1.97 |
| APL_0904 | *kdkA* | 3-deoxy-D-manno-octulosonic acid kinase | -1.99 |
| APL_2035 | *sufI* | Copper-containing nitrite reductase precursor | -1.99 |
| APL_0607 | *nfnB* | putative NAD(P)H nitroreductase | -1.99 |
| APL_1556 |  | transport system permease protein | -1.99 |
| APL_1218 | *fur* | ferric uptake regulation protein | -2 |
| APL_0048 | *arcA* | aerobic respiration control protein ArcA | -2 |
| APL_1819 | *acpP* | acyl carrier protein | -2.01 |
| APL_1148 |  | periplasmic protein | -2.01 |
| APL_0914 | *ppiB* | peptidyl-prolyl cis-trans isomerase B | -2.01 |
| APL_0752 | *seqA* | DNA replication inhibitor protein | -2.01 |
| APL_2040 | *kdsA* | 2-dehydro-3-deoxyphosphooctonate aldolase | -2.01 |
| APL_0093 |  | hypothetical protein | -2.02 |
| APL_1227 |  | hypothetical protein | -2.02 |
| APL_0844 | *queF* | NADPH-dependent 7-cyano-7-deazaguanine reductase | -2.03 |
| APL_0336 | *yhgB* | ABC transporter ATP-binding prote | -2.03 |
| APL_0471 |  | hypothetical protein | -2.05 |
| APL_1506 |  | LysM domain/BON superfamily protein | -2.05 |
| APL_0408 | *fabZ* | (3R)-hydroxymyristoyl-[acyl-carrier-protein] dehydratase | -2.06 |
| APL_0812 | *res* | type III restriction enzyme | -2.06 |
| APL_1230 | *serB* | phosphoserine phosphatase | -2.07 |
| APL_2002 |  | hypothetical protein | -2.08 |
| APL_1908 | *xylA* | Xylose isomerase | -2.1 |
| APL_0896 | *fdhE* | formate dehydrogenase accessory protein | -2.1 |
| APL_1793 |  | Fe(III) dicitrate ABC transporter, ATP-binding protein | -2.1 |
| APL_0698 | *argA* | amino-acid acetyltransferase | -2.11 |
| APL_0087 | *nudC* | NADH pyrophosphatase | -2.12 |
| APL_1045 |  | membrane protein | -2.14 |
| APL_1379 | *ccp* | cytochrome c peroxidase | -2.15 |
| APL_2039 | *ldhA* | Glycerate dehydrogenase | -2.15 |
| APL_1591 |  | hypothetical protein | -2.16 |
| APL_0630 | *mazG* | pyrophosphatase | -2.16 |
| APL_1594 | *lipB* | lipoyltransferase | -2.17 |
| APL_1147 | *trpG* | anthranilate synthase component II | -2.17 |
| APL_0547 | *tadF* | tight adherence protein F | -2.18 |
| APL_1078 | *trxA* | thioredoxin | -2.18 |
| APL_1098 |  | 6-pyruvoyl tetrahydrobiopterin synthase | -2.18 |
| APL_1495 |  | transcriptional regulator | -2.18 |
| APL_0130 | *engC* | GTPase EngC | -2.19 |
| APL_1070 | *ftnB* | ferritin-like protein 2 | -2.2 |
| APL_0220 |  | lipoprotein | -2.2 |
| APL_0923 |  | hypothetical protein | -2.21 |
| APL_0270 |  | hypothetical protein | -2.21 |
| APL_0975 |  | hypothetical protein | -2.22 |
| APL_1642 | *tusD* | sulfurtransferase TusD-like protein | -2.22 |
| APL_0205 |  | predicted rRNA methyltransferase | -2.26 |
| APL_0717 |  | iron(III) ABC transporter, ATP-binding protein | -2.27 |
| APL_0715 |  | ABC transport system permease protein | -2.27 |
| APL_0054 | *grxA* | glutaredoxin | -2.28 |
| APL_0583 |  | hypothetical protein | -2.3 |
| APL_1853 | *ilvC* | Ketol-acid reductoisomerase | -2.32 |
| APL_0392 |  | hypothetical protein | -2.32 |
| APL_1795 |  | periplasmic iron/siderophore binding protein | -2.32 |
| APL_0202 | *thiL* | Thiamine-monophosphate kinase | -2.34 |
| APL_1160 | *rluB* | ribosomal large subunit pseudouridine synthase B | -2.35 |
| APL_0457 |  | metalloprotease | -2.35 |
| APL_0334 |  | hypothetical protein | -2.36 |
| APL_1732 |  | hypothetical protein | -2.36 |
| APL_0836 |  | transcriptional regulator | -2.37 |
| APL_1949 | *dtd* | D-tyrosyl-tRNA(Tyr) deacylase | -2.38 |
| APL_0714 |  | ABC transport system periplasmic protein | -2.38 |
| APL_1578 | *kdsA* | 2-dehydro-3-deoxyphosphooctonate aldolase | -2.38 |
| APL_1516 |  | cytochrome c biogenesis factor-like protein | -2.39 |
| APL_0337 |  | hypothetical protein | -2.41 |
| APL_0190 | *fis* | DNA-binding protein Fis | -2.41 |
| APL_0276 | *frpB* | iron-regulated outer membrane protein B | -2.42 |
| APL_0864 |  | hypothetical protein | -2.45 |
| APL_1005 | *rpsO* | 30S ribosomal protein S15 | -2.45 |
| APL_0326 | *prfB* | peptide chain release factor 2 | -2.46 |
| APL_1274 |  | hypothetical protein | -2.47 |
| APL_0293 |  | type I site-specific restriction-modification system, R (restriction) subunit | -2.49 |
| APL_0863 |  | hypothetical protein | -2.49 |
| APL_0363 |  | hypothetical protein (coproporphyrinogen III oxidase) | -2.5 |
| APL_2003 |  | hypothetical protein | -2.54 |
| APL_1957 |  | Lipoprotein_5 domain containing protein | -2.54 |
| APL_0605 |  | hypothetical protein | -2.56 |
| APL_1595 |  | hypothetical protein | -2.58 |
| APL_0709 |  | hypothetical protein | -2.58 |
| APL_0450 | *mglB* | D-galactose-binding periplasmic protein precursor | -2.59 |
| APL_0840 | *tolC* | outer membrane protein | -2.59 |
| APL_1810 |  | Mg-dependent DNAse | -2.59 |
| APL_0073 | *ydhD* | monothiol glutaredoxin-like protein | -2.6 |
| APL_0125 |  | lipoprotein | -2.62 |
| APL_1972 | *rpmG* | 50S ribosomal protein L33 | -2.63 |
| APL_0253 | *recD* | exodeoxyribonuclease V alpha chain | -2.64 |
| APL_0862 | *truC* | tRNA pseudouridine synthase C | -2.64 |
| APL_1231 |  | nucleotide-binding protein | -2.64 |
| APL_0290 |  | Type I restriction enzyme EcoR124II specificity | -2.65 |
| APL_0227 |  | lipoprotein | -2.65 |
| APL_1087 | *clpB* | chaperone ClpB | -2.65 |
| APL_0443 |  | autotransporter adhesin | -2.66 |
| APL_1917 | *metJ* | transcriptional repressor protein MetJ | -2.69 |
| APL_0959 |  | hemagglutinin/hemolysin-like protein | -2.7 |
| APL_1658 |  | hypothetical protein | -2.7 |
| APL_0366 |  | hypothetical protein | -2.7 |
| APL_1922 | *pgaB* | Biofilm PGA synthesis lipoprotein PgaB precursor | -2.71 |
| APL_1818 |  | hypothetical protein | -2.72 |
| APL_0124 |  | hypothetical protein | -2.73 |
| APL_1707 |  | hypothetical protein | -2.73 |
| APL_0264 |  | putative ABC transporter ATP-binding protein | -2.74 |
| APL_1836 |  | hypothetical protein | -2.77 |
| APL_0376 | *lon* | ATP-dependent protease La | -2.77 |
| APL_0631 | *accD* | acetyl-coenzyme A carboxylase carboxyl transferase subunit beta | -2.78 |
| APL_1804 |  | hypothetical protein | -2.78 |
| APL_0622 | *tdk* | thymidine kinase | -2.79 |
| APL_1726 |  | hypothetical protein | -2.79 |
| APL_0059 | *narP* | nitrate/nitrite response regulator protein | -2.8 |
| APL_0285 | *ubiG* | 3-demethylubiquinone-9 3-methyltransferase | -2.82 |
| APL_0395 | *rseA* | sigma-E factor negative regulatory protein | -2.83 |
| APL_1033 | *pheA* | P-protein | -2.84 |
| APL_0335 | *ptsN* | PTS system, nitrogen regulatory IIA-like protein | -2.84 |
| APL_0670 |  | Fe2+/Pb2+ permease | -2.85 |
| APL_1816 | *holB* | DNA polymerase III subunit delta | -2.86 |
| APL_1657 |  | amino-acid ABC transporter-binding protein | -2.93 |
| APL_1285 |  | hypothetical protein | -2.93 |
| APL_0126 |  | HIT-like protein | -2.94 |
| APL_0078 | *exbB2* | biopolymer transport protein ExbB2 | -2.97 |
| APL_1047 | *hgbA* | hemoglobin-binding protein A precursor | -2.97 |
| APL_0784 |  | hypothetical protein | -2.98 |
| APL_1904 |  | hypothetical protein | -2.98 |
| APL_1921 | *pgaA* | biofilm PGA synthesis protein PgaA precursor | -3.03 |
| APL_1794 |  | pseudoazurin | -3.04 |
| APL_0937 |  | hypothetical protein | -3.09 |
| APL_0694 |  | hypothetical protein | -3.1 |
| APL_1378 | *phnA* | alkylphosphonate uptake protein | -3.11 |
| APL_0458 | *dsbC* | thiol:disulfide interchange protein DsbC precursor | -3.12 |
| APL_1257 | *phoB* | phosphate regulon transcriptional regulatory protein PhoB | -3.14 |
| APL_1597 |  | rare lipoprotein A RlpA-like protein | -3.14 |
| APL_2042 | *hemK* | HemK-like protein | -3.14 |
| APL_1211 |  | hypothetical protein | -3.15 |
| APL_1705 |  | FKBP-type peptidyl-prolyl cis-trans isomerase | -3.15 |
| APL_1869 |  | hypothetical protein | -3.25 |
| APL_0643 |  | hypothetical protein | -3.26 |
| APL_1210 |  | hypothetical protein | -3.3 |
| APL_0590 |  | hypothetical protein (Exopolysaccharide biosynthesis protein) | -3.32 |
| APL_0459 | *recJ* | single-stranded-DNA-specific exonuclease RecJ | -3.32 |
| APL_0236 |  | lipoprotein | -3.34 |
| APL_0563 | *afuA_2* | Fe3+ ABC transporter, iron-binding protein | -3.36 |
| APL_1736 | *hslV* | ATP-dependent protease | -3.44 |
| APL_2041 |  | hypothetical protein | -3.47 |
| APL_0049 |  | hypothetical protein | -3.48 |
| APL_0149 |  | hypothetical protein | -3.52 |
| APL_0705 |  | methylation subunit, type III restriction-modification system | -3.53 |
| APL_1735 | *hslU* | ATP-dependent hsl protease ATP-binding subunit | -3.54 |
| APL_1289 |  | hypothetical protein | -3.54 |
| APL_1432 |  | putative NAD(P)H oxidoreductase | -3.59 |
| APL_0391 | *macA* | macrolide-specific efflux protein | -3.61 |
| APL_1139 | *aroE* | shikimate dehydrogenase | -3.61 |
| APL_0113 |  | hypothetical protein | -3.62 |
| APL_1159 |  | lipoprotein | -3.7 |
| APL_0077 | *exbD2* | biopolymer transport protein ExbD2 | -3.7 |
| APL_1350 | *tehB* | tellurite resistance protein TehB-like protein | -3.7 |
| APL_0114 | *hupA* | DNA-binding protein HU | -3.72 |
| APL_1570 | *exbB* | biopolymer transport ExbB protein | -3.75 |
| APL_1141 |  | hypothetical protein | -3.76 |
| APL_1802 |  | hypothetical protein | -3.78 |
| APL_1569 | *exbD* | biopolymer transport ExbD protein | -3.8 |
| APL_0418 |  | hypothetical protein | -3.8 |
| APL_1817 | *tmk* | thymidylate kinase | -3.81 |
| APL_1815 |  | hypothetical protein | -3.82 |
| APL_0394 | *rpoE* | RNA polymerase sigma-70 factor | -3.84 |
| APL_0495 |  | putative DNA-methyltransferase | -3.84 |
| APL_0946 |  | hypothetical protein | -3.91 |
| APL_1140 |  | hypothetical protein | -3.93 |
| APL_0407 | *lpxA* | Acyl-[acyl-carrier-protein]--UDP-N-acetylglucosamine O-acyltransferase | -3.94 |
| APL_0440 |  | hypothetical protein | -4.03 |
| APL_1142 | *recX* | regulatory protein RecX | -4.15 |
| APL_0134 |  | hypothetical protein | -4.21 |
| APL_0428 | *smpA* | small protein A | -4.27 |
| APL_1143 | *recA* | recombinase A | -4.34 |
| APL_1791 |  | periplasmic iron/siderophore binding protein | -4.7 |
| APL_0076 | *tonB* | protein TonB2 | -4.89 |
| APL_0086 |  | hypothetical protein | -4.91 |
| APL_0259 |  | oligoketide cyclase/lipid transport protein | -5.03 |
| APL_0426 |  | hypothetical protein | -5.16 |
| APL_1643 |  | hypothetical protein | -5.32 |
| APL_0901 |  | ribosome-associated inhibitor A | -5.42 |
| APL_0330 |  | lipoprotein | -5.73 |
| APL_1281 |  | hypothetical protein | -5.92 |
| APL_0623 |  | hypothetical protein | -6.11 |
| APL_0442 | *sanA* | SanA protein | -6.25 |
| APL_0987 | *htpG* | chaperone protein HtpG | -6.32 |
| APL_0668 |  | hypothetical protein | -6.9 |
| APL_1644 |  | hypothetical protein | -7.04 |
| APL_0559 | *flp1* | fimbrial protein Flp precursor | -7.1 |
| APL_2031 |  | hypothetical protein | -7.83 |
| APL_1891 |  | hypothetical protein | -8.18 |
| APL_1299 |  | TonB dependent/Ligand-Gated channel | -8.74 |
| APL_1875 |  | lipoprotein | -8.88 |
| APL_1228 | *infA* | translation initiation factor IF-1 | -9.69 |

**Table S5. Genes that were significantly (FDR=0) up-regulated or down-regulated in a 28h biofilm cultured in a drip-flow apparatus (biofilm vs plankton)**.

| **Locus tag** | **Gene** | **Description** | **Fold change** |
| --- | --- | --- | --- |
| APL_0225 | *rplT* | 50S ribosomal protein L20 | 4.05 |
| APL_1785 | *rplQ* | 50S ribosomal protein L17 | 3.91 |
| APL_0189 | *dus* | tRNA-dihydrouridine synthase B | 3.82 |
| APL_0449 |  | integral membrane protein | 3.71 |
| APL_0740 | *rpsA* | 30S ribosomal protein S1 | 3.56 |
| APL_1759 | *rpsJ* | 30S ribosomal protein S10 | 3.46 |
| APL_1789 | *rplS* | 50S ribosomal protein L19 | 3.34 |
| APL_1962 | *hflX* | GTP-binding protein hflX | 3.32 |
| APL_1020 | *uxaC* | uronate isomerase | 3.21 |
| APL_1721 | *rplL* | 50S ribosomal protein L7/L12 | 3.19 |
| APL_0487 | *rplY* | 50S ribosomal protein L25 | 3.16 |
| APL_1171 | *rpsF* | 30S ribosomal protein S6 | 3.15 |
| APL_1387 |  | hypothetical protein (metal-binding/nucleic acid-binding protein) | 3.07 |
| APL_1782 | *rpsK* | 30S ribosomal protein S11 | 3.02 |
| APL_1319 | *ptsB* | PTS system sucrose-specific EIIBC component | 2.98 |
| APL_1991 |  | Branched-chain amino acid transport system carrier protein braB (Branched-chain amino acid uptake carrier braB) | 2.96 |
| APL_1169 | *rplI* | 50S ribosomal protein L9 | 2.94 |
| APL_1769 | *rpsQ* | 30S ribosomal protein S17 | 2.9 |
| APL_1781 | *rpsM* | 30S ribosomal protein S13 | 2.81 |
| APL_2029 |  | membrane protein | 2.81 |
| APL_0297 | *cydA* | cytochrome oxidase subunit 1 | 2.5 |
| APL_0191 |  | Na+-dependent transporters of the SNF family | 2.5 |
| APL_0226 |  | hypothetical protein (RecA-superfamily ATPases implicated in signal transduction) | 2.47 |
| APL_0527 |  | ABC-type transport system, permease component | 2.44 |
| APL_1670 | *rbsA* | ribose transport ATP-binding protein RbsA | 2.42 |
| APL_1783 | *rpsD* | 30S ribosomal protein S4 | 2.4 |
| APL_0977 | *xthA* | exodeoxyribonuclease III | 2.4 |
| APL_0767 | *sst* | serine/threonine transporter | 2.38 |
| APL_0201 | *nusB* | transcription antitermination protein | 2.36 |
| APL_1222 |  | aspartate-semialdehyde dehydrogenase | 2.36 |
| APL_1016 |  | nucleoside transporter | 2.35 |
| APL_0735 |  | hypothetical protein | 2.34 |
| APL_0936 |  | membrane protein | 2.34 |
| APL_0811 |  | hypothetical protein | 2.33 |
| APL_2016 |  | Ferrioxamine B receptor precursor | 2.26 |
| APL_0659 | *purE* | phosphoribosylaminoimidazole carboxylase catalytic subunit | 2.26 |
| APL_1772 | *rpsN* | 30S ribosomal protein S14 | 2.26 |
| APL_1762 | *rplW* | 50S ribosomal protein L23 | 2.25 |
| APL_1215 |  | ABC transporter ATP-binding protein | 2.24 |
| APL_0382 | *ribD* | riboflavin biosynthesis protein | 2.23 |
| APL_0982 | *rpmE* | 50S ribosomal protein L31 | 2.23 |
| APL_1150 | *ntpA* | dATP pyrophosphohydrolase | 2.22 |
| APL_0850 |  | ABC transporter, ATP-binding/permease | 2.22 |
| APL_0343 | *fruA* | PTS system fructose-specific EIIBC component | 2.17 |
| APL_1823 | *pbpB* | penicillin-binding protein 1B (PBP1b) | 2.16 |
| APL_0173 |  | hypothetical protein | 2.15 |
| APL_0708 | *ppx* | exopolyphosphatase | 2.15 |
| APL_0682 | *hpt* | hypoxanthine phosphoribosyltransferase | 2.15 |
| APL_0345 | *fruB* | multiphosphoryl transfer protein | 2.14 |
| APL_0321 | *dsbB* | disulfide bond formation protein B | 2.13 |
| APL_0182 | *dnaQ* | DNA polymerase III, epsilon subunit and related 3-5 exonucleases | 2.12 |
| APL_1558 | *psT* | 30S ribosomal protein S20 | 2.11 |
| APL_0718 |  | hypothetical protein | 2.1 |
| APL_1443 | *apxIB* | toxin RTX-I translocation ATP-binding protein | 2.07 |
| APL_0847 |  | oxygen-independent coproporphyrinogen III oxidase-like protein | 2.02 |
| APL_1105 | *dnaE* | DNA polymerase III subunit alpha | 2.02 |
| APL_0181 | *gloA* | lactoylglutathione lyase | 2 |
| APL_0099 | *ilvG* | Acetolactate synthase isozyme II large subunit (AHAS-II) | 1.99 |
| APL_0344 | *fruK* | 1-phosphofructokinase | 1.96 |
| APL_1367 | *ccmF* | cytochrome c-type biogenesis protein | 1.95 |
| APL_0568 |  | hypothetical protein | 1.95 |
| APL_2012 |  | membrane protein | 1.93 |
| APL_0640 | *rbfA* | ribosome-binding factor A | 1.92 |
| APL_1474 | *dnaG* | DNA primase | 1.91 |
| APL_1110 |  | membrane protein | 1.9 |
| APL_1370 | *ccmC* | heme exporter protein C | 1.89 |
| APL_1660 |  | mannose permease IID component | 1.88 |
| APL_1926 | *truD* | tRNA pseudouridine synthase D | 1.86 |
| APL_1495 |  | transcriptional regulator | -1.89 |
| APL_0336 | *yhgB* | ABC transporter ATP-binding prote | -1.92 |
| APL_1735 | *hslU* | ATP-dependent hsl protease ATP-binding subunit | -1.95 |
| APL_0293 |  | type I site-specific restriction-modification system, R (restriction) subunit | -1.98 |
| APL_0391 | *macA* | macrolide-specific efflux protein | -2 |
| APL_0626 | *macB* | macrolide-specific ABC-type efflux carrier | -2.02 |
| APL_0093 |  | hypothetical protein | -2.02 |
| APL_1707 |  | hypothetical protein | -2.04 |
| APL_1211 |  | hypothetical protein | -2.04 |
| APL_0234 |  | lipoprotein | -2.06 |
| APL_0205 |  | predicted rRNA methyltransferase | -2.07 |
| APL_0334 |  | hypothetical protein | -2.08 |
| APL_0629 | *cpxR* | transcriptional regulatory protein CpxR | -2.08 |
| APL_0062 | *mipB* | transaldolase | -2.09 |
| APL_0840 | *tolC* | outer membrane protein | -2.11 |
| APL_1425 | *napC* | cytochrome c-type protein | -2.13 |
| APL_1597 |  | rare lipoprotein A RlpA-like protein | -2.13 |
| APL_0614 | *bioD1* | dethiobiotin synthetase 1 | -2.15 |
| APL_1159 |  | lipoprotein | -2.18 |
| APL_0163 |  | anaerobic ribonucleoside triphosphate reductase | -2.23 |
| APL_1045 |  | membrane protein | -2.23 |
| APL_0331 | *hlp* | lipoprotein | -2.24 |
| APL_2019 | *hisG* | ATP phosphoribosyltransferase (ATP-PRTase) (ATP-PRT) | -2.25 |
| APL_1815 |  | hypothetical protein | -2.27 |
| APL_1136 | *amiB* | N-acetylmuramoyl-L-alanine amidase AmiB precursor | -2.27 |
| APL_1379 | *ccp* | cytochrome c peroxidase | -2.27 |
| APL_0630 | *mazG* | pyrophosphatase | -2.28 |
| APL_1791 |  | periplasmic iron/siderophore binding protein | -2.31 |
| APL_0643 |  | hypothetical protein | -2.32 |
| APL_1513 | *coaA* | pantothenate kinase | -2.32 |
| APL_1640 | *fkpA* | putative FKBP-type peptidyl-prolyl cis-trans isomerase | -2.33 |
| APL_0623 |  | hypothetical protein | -2.36 |
| APL_0694 |  | hypothetical protein | -2.36 |
| APL_1817 | *tmk* | thymidylate kinase | -2.39 |
| APL_0113 |  | hypothetical protein | -2.39 |
| APL_1697 |  | hypothetical protein | -2.39 |
| APL_1658 |  | hypothetical protein | -2.4 |
| APL_0236 |  | lipoprotein | -2.42 |
| APL_0333 | *visC* | monooxygenase family protein | -2.42 |
| APL_1641 |  | hypothetical protein | -2.47 |
| APL_0392 |  | hypothetical protein | -2.48 |
| APL_1228 | *infA* | translation initiation factor IF-1 | -2.49 |
| APL_2003 |  | hypothetical protein | -2.51 |
| APL_0339 | *pepC* | phosphoenolpyruvate carboxylase | -2.52 |
| APL_1285 |  | hypothetical protein | -2.53 |
| APL_1726 |  | hypothetical protein | -2.53 |
| APL_1736 | *hslV* | ATP-dependent protease | -2.55 |
| APL_0394 | *rpoE* | RNA polymerase sigma-70 factor | -2.59 |
| APL_1832 |  | hypothetical protein | -2.59 |
| APL_1516 |  | cytochrome c biogenesis factor-like protein | -2.59 |
| APL_0330 |  | lipoprotein | -2.61 |
| APL_0338 |  | hypothetical protein | -2.62 |
| APL_0705 |  | methylation subunit, type III restriction-modification system | -2.67 |
| APL_1143 | *recA* | recombinase A | -2.68 |
| APL_0590 |  | hypothetical protein (Exopolysaccharide biosynthesis protein) | -2.68 |
| APL_1842 | *cysI* | Sulfite reductase [NADPH] hemoprotein beta-component (SIR-HP) (SIRHP) | -2.71 |
| APL_0400 | *surA* | peptidyl-prolyl cis-trans isomerase SurA | -2.77 |
| APL_1167 |  | hypothetical protein | -2.78 |
| APL_0149 |  | hypothetical protein | -2.91 |
| APL_1869 |  | hypothetical protein | -3.06 |
| APL_1856 |  | hypothetical protein | -3.12 |
| APL_0800 | *pckA* | phosphoenolpyruvate carboxykinase (ATP) | -3.12 |
| APL_2031 |  | hypothetical protein | -3.19 |
| APL_1837 | *asnA* | aspartate-ammonia ligase | -3.26 |
| APL_1642 | *tusD* | sulfurtransferase TusD-like protein | -3.26 |
| APL_0248 | *cysK* | cysteine synthase | -3.27 |
| APL_1857 | *merP* | MerP | -3.27 |
| APL_0450 | *mglB* | D-galactose-binding periplasmic protein precursor | -3.31 |
| APL_0946 |  | hypothetical protein | -3.5 |
| APL_1836 |  | hypothetical protein | -3.53 |
| APL_0132 |  | haloacid dehalogenase-like hydrolase | -3.61 |
| APL_0285 | *ubiG* | 3-demethylubiquinone-9 3-methyltransferase | -3.66 |
| APL_1420 | *mglB* | D-galactose-binding periplasmic protein precursor | -3.74 |
| APL_1855 |  | hypothetical protein | -3.81 |
| APL_1714 | *ulaA* | ascorbate-specific permease IIC component UlaA | -3.87 |
| APL_1904 |  | hypothetical protein | -3.9 |
| APL_0378 | *glpQ* | glycerophosphoryl diester phosphodiesterase | -3.92 |
| APL_1957 |  | Lipoprotein_5 domain containing protein | -4.03 |
| APL_0252 | *alr* | alanine racemase | -4.03 |
| APL_1657 |  | amino-acid ABC transporter-binding protein | -4.09 |
| APL_0615 | *mlc* | transcriptional repressor of carbohydrate metabolism | -4.19 |
| APL_1446 | *afuA* | ABC-type Fe3+ transport system, periplasmic component | -4.38 |
| APL_1891 |  | hypothetical protein | -4.38 |
| APL_1289 |  | hypothetical protein | -4.52 |
| APL_1494 | *ftpA* | fine tangled pili major subunit | -4.66 |
| APL_0375 | *glpK* | glycerol kinase | -4.71 |
| APL_0563 | *afuA_2* | Fe3+ ABC transporter, iron-binding protein | -4.97 |
| APL_1432 |  | putative NAD(P)H oxidoreductase | -5.09 |
| APL_1694 |  | antigenic protein, ABC transporter-like protein | -5.43 |
| APL_1875 |  | lipoprotein | -5.54 |
| APL_1644 |  | hypothetical protein | -6.01 |
| APL_0668 |  | hypothetical protein | -8.19 |
| APL_0901 |  | ribosome-associated inhibitor A | -9.28 |
